# Two New Iridoid Glucosides from the Whole Plant of *Patrinia scabiosifolia* Link

**DOI:** 10.3390/molecules26144201

**Published:** 2021-07-10

**Authors:** Shikai Sun, Jiang Fu, Kaidong Liu, Mingzhu Dai, Yong Li, Yunbao Liu, Shuanggang Ma, Jing Qu

**Affiliations:** State Key Laboratory of Bioactive Substance and Function of Natural Medicines, Institute of Materia Medica, Chinese Academy of Medical Sciences, Peking Union Medical College, Beijing 100050, China; sun_shikai@126.com (S.S.); fujiang@imm.ac.cn (J.F.); liukaidong@imm.ac.cn (K.L.); daimingzhu@imm.ac.cn (M.D.); liyong@imm.ac.cn (Y.L.); liuyunbao@imm.ac.cn (Y.L.); mashuanggang@imm.ac.cn (S.M.)

**Keywords:** *Patrinia scabiosifolia* Link, iridoid glucosides, anti-inflammatory

## Abstract

As a traditional Chinese medicine, *Patrinia scabiosifolia* Link has been used to treat various inflammatory-related diseases, and recent studies have shown that it possesses potent anti-inflammatory activity. Therefore, phytochemical investigation on whole plants of *P. scabiosifolia* were carried out, which led to the isolation of two new iridoid glucosides, patriniscabiosides A (**1**) and B (**2**), together with six known compounds (**3**–**8**). The structural elucidation of all compounds was performed by HRESIMS and extensive spectroscopic analyses including IR, 1D, 2D NMR, and electronic circular dichroism (ECD). All the isolated compounds were tested for their anti-inflammatory activity using the NF-κB-Dependent Reporter Gene Expression Assay, and compound **3** displayed anti-inflammatory activity through the inhibition of the NF-κB pathway, with an inhibitory rate of 73.44% at a concentration of 10 μM.

## 1. Introduction

Growing evidence supports the idea that inflammation plays an important role in various human diseases including cerebrovascular, cardiovascular, pulmonary, blood, liver, and intestinal diseases [1]. Nuclear transcription factor κB (NF-κB) is an important transcription factor which regulates the expression of a variety of genes involved in immune and inflammatory responses, and the activation of the NF-κB pathway can lead to the induction of many inflammatory cytokines [2]. Therefore, compounds targeting the NF-κB signaling pathway are considered promising anti-inflammatory agents.

Iridoids, a large and still expanding class of monoterpenoids, are a type of secondary metabolites that can be found in many folk medicinal plants. Recent studies revealed that iridoids exhibit a wide range of bioactivities, including anti-inflammatory [3,4,5], antitumor [6], neuroprotective [7,8,9], and hepatoprotective effects [10,11], and can serve as promising lead compounds in drug discovery.

As an important source of iridoids, *Patrinia scabiosifolia* Link, a perennial plant belonging to the Caprifoliaceae family, is distributed widely in East Asia including China, Japan, and North Korea [12]. The whole plants of *P. scabiosifolia* have been used as traditional Chinese medicine to treat appendicitis, diarrhea, and postpartum abdominal pain [13]. Previous phytochemical investigations on *P. scabiosifolia* led to the isolation of triterpenoids [14,15,16,17,18,19,20,21,22], iridoids [21,22,23,24,25], flavonoids [21], and lignans [26]. 

**Figure 1:**
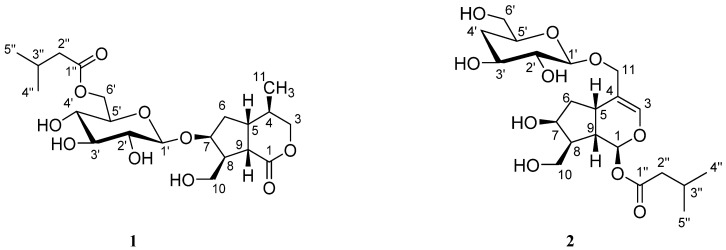
Structures of compounds **1** and **2**.

As part of an ongoing project to search for novel bioactive compounds, the whole plants of *P. scabiosifolia* was further investigated, leading to the isolation of two new iridoid glucosides (**1**, **2**) (Figure 1) and six known iridoids and iridoid glucosides (**3**~**8**). Compound **3** was shown to possess anti-inflammatory activity by the inhibition of the NF-κB pathway. Herein, we describe the isolation, structure elucidation, and pharmacological evaluation of these compounds.

## 2. Results and Discussions

### 2.1. Chemical Structure of Compound ***1***

Compound **1** was obtained as a white powder. Its molecular formula (C_21_H_34_O_10_) was assigned by HRESIMS, which showed an [M + H]^+^ ion peak at *m/z* 447.22247 (calcd for C_21_H_35_O_10_, 447.22247), indicating five degrees of unsaturation. The IR spectrum indicated the presence of hydroxyl (3383 cm^−^^1^) and carbonyl (1733 cm^−1^) groups. The ^1^H-NMR spectrum (Table 1) displayed three oxygenated methylene groups at *δ*_H_ 4.47 (1H, dd, *J* = 11.8, 2.1 Hz, H-6′a), 4.14 (1H, dd, *J* = 11.8, 5.8 Hz, H-6′b), 4.14 (1H, dd, *J* = 10.7, 3.2 Hz, H-3a), 3.97 (1H, t, *J* = 10.7 Hz, H-3b), 3.89 (1H, dd, *J* = 11.0, 4.8 Hz, H-10a), and 3.82 (1H, dd, *J* = 11.0, 9.0 Hz, H-10b), two oxygenated methines at *δ*_H_ 4.39 (1H, d, *J* = 7.8 Hz, H-1′), 4.26 (1H, br.t, *J* = 3.8 Hz, H-7), and three methyl groups at *δ*_H_ 0.95 (6H, d, *J* = 6.7 Hz, Me-4″, Me-5″) and 0.99 (3H, d, *J* = 6.7 Hz, Me-11). Analysis of the ^13^C-NMR spectrum revealed 21 carbon signals, including two carbonyls (*δ*c 178.1, 174.5), six oxygenated tertiary carbons (*δ*c 105.3, 83.7, 77.9, 75.4, 75.3, 71.5), three oxygenated methylenes (*δ*c 74.4, 64.3, 61.8), five methines (*δ*c 51.3, 43.3, 43.1, 37.3, 27.0), two methylenes (*δ*c 44.3, 40.5), and three methyls (*δ*c 22.8, 22.8, 15.8).

**Figure 2:**
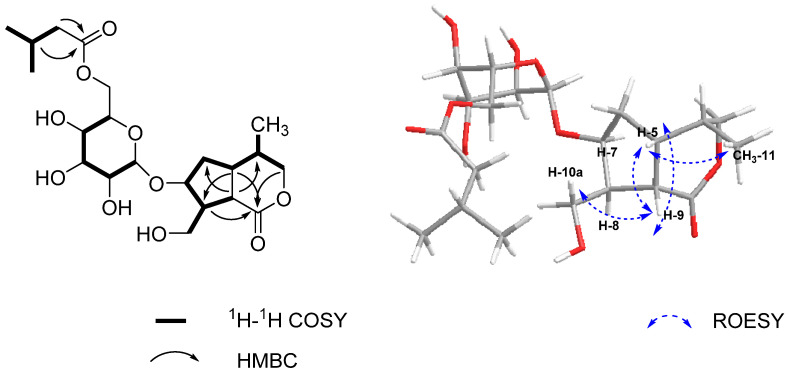
Key ^1^H-^1^H COSY, HMBC, and ROESY correlations for compound **1**.

^1^H-^1^H COSY spectrum (Figure 2), in combination with HSQC and NMR data, indicated three coupling systems: the correlations H-3/H-4, H-4/H-5, H-4/Me-11, H-5/H-6, H-6/H-7, H-7/H-8, and H-8/H-9, H-8/H_2_-10 revealed a cyclopentane iridoid skeleton involving C(3)H_2_–C(4)H–C(5)H–C(6)H_2_–C(7)H–C(8)H–C(9)H, which was further confirmed by HMBC correlations from H-9 to C-4, C-6, from H-5 to C-1, C-8, and from H-3, H-5, H-8 to C-1; the correlations H-1′/H-2′, H-2′/H-3′, H-3′/H-4′, H-4′/H-5′, and H-5′/H-6′, in combination with ^13^C-NMR data, indicated a characteristic glucopyranosyl unit, whose configuration was determined to be *β* based on the coupling constants (*J* = 7.8 Hz) of the anomeric proton; an isovaleryl ester moiety was established by the ^1^H-^1^H COSY correlations H-3″/H-2″, H-3″/H-4″, and H-3″/H-5″, which was supported by HMBC correlations from H-2″, H-3″ to C-1″.

The connection between the three fragments could be further determined by the HMBC spectrum. The correlation from H-1′ to C-7 and from H-7 to C-1′ indicated that the glucopyranosyl unit was attached to 7-OH, which was also supported by the downfield chemical shift of C-7 (*δ*c 83.7). The correlation from H-6′ to C-1″ determined that the isovaleryl ester moiety was attached to 6′-OH. Thus, the planar structure of **1** was established.

The relative configuration of **1** was assigned on the basis of the ROESY spectrum. The correlations H-5/Me-11 and H-5/H-9 indicated that H-5, H-9, and Me-11 were on the same face of the ring, while the correlation H-9/H10 determined H-8 to be on the other face. Meanwhile, the correlation H-7/H-8 in combination with the absence of correlation fpr H-7/H-5, H-7/H-9, H-7/H-10b suggested that H-7 was on the same face as H-8.

The absolute configuration of **1** was further confirmed by electronic circular dichroism (ECD) (Figure S10, See Supplementary Materials), which showed a negative Cotton effect at 217.5 nm [27], suggesting the absolute configuration to be 4*R*,5*R*,7*S*,8*S*,9*S*. Thus, the structure of compound **1** was confirmed, as shown in Figure 1, and the compound was named patriniscabioside A.

### 2.2. Chemical Structure of Compound ***2***

**Figure 3:**
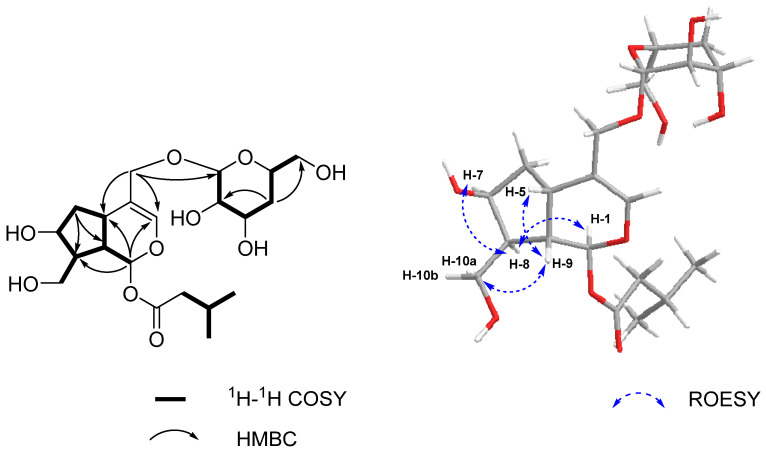
Key ^1^H-^1^H COSY, HMBC, and ROESY correlations for compound **2**.

Compound **2** was obtained as a yellow oil and was assigned the molecular formula C_21_H_34_O_10_, as deduced from the [M + HCOO]^−^ ion at *m/z* 491.21338 (calcd for C_22_H_35_O_12_ 491.21340). The IR spectrum suggested the presence of hydroxyl (3377 cm^−1^) and carbonyl (1747 cm^−1^) groups. The ^1^H and ^13^C NMR data of **2** (Table 1) resembled those reported for patrinoside [28], except for the sugar moiety. The analysis of the molecular formula, in combination with the chemical shift *δ*c 36.4 of **2,** suggested the presence of a deoxysaccharide unit. The ^1^H-^1^H COSY correlation (Figure 3) between H-4″ (*δ*_H_ 1.92, 1.36) and H-3″ (*δ*_H_ 3.60), H-5″ (*δ*_H_ 3.53) revealed that deoxygenation occurred at the C-4″ position, which was further supported by the HMBC correlation from H-2″ (*δ*_H_ 3.10), H-6″ (*δ*_H_ 3.57) to C-4″ (*δ*c 36.4). Thus, a planar structure was established.

**Figure 4:**
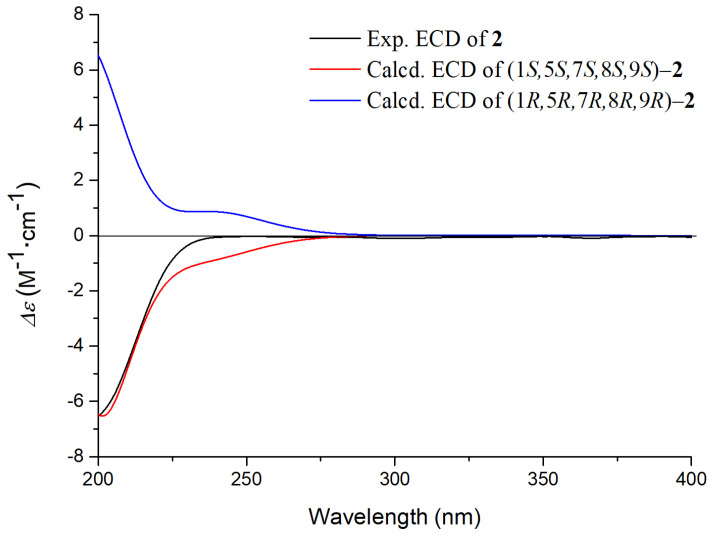
Calculated and experimental ECD for compound **2**.

Additionally, the ROESY correlations H-1/H-8, H-5/H-9, H-9/H-10a indicated that H-1, H-8 were on the same side, while H-5, H-9 were on the other side. Furthermore, the ROESY correlation H-7/H-8, together with the absence of correlation between H-7/H-5, H-9 and H-10b suggested that H-7 was also on the same side as H-8. To further determine the absolute configuration of **2**, ECD calculation was conducted using time-dependent density functional theory (TD-DFT) at the B3LYP/6-31+G(d,p) level. The calculated ECD spectrum of (1*S*,5*S*,7*S*,8*S*,9*S*) model displayed a negative Cotton effect at 200 nm, which was in agreement with the experimental ECD of compound **2** (Figure 4). Thus, compound **2** was characterized as shown and named patriniscabioside B.

### 2.3. Chemical Structures of Compounds ***3***–***8***

**Figure 5:**
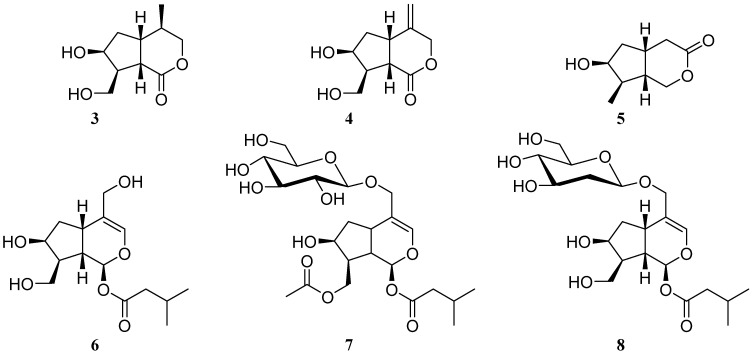
Structures of compounds **3**–**8**.

The known compounds were identified as (4*R*,5*R*,7*S*,8*S*,9*S*)-7-hydroxy-8-hydroxymethyl-4-methyl-perhydrocyclopenta[*c*]pyran-1-one (**3**) [29], 6-hydroxy-7-(hydroxymethyl)-4-methylenehexahydrocyclopenta[*c*]pyran-1(3*H*)-one (**4**) [30], 6-hydroxy-7-methylhexahydrocyclopenta[*c*]pyran-3-one (**5**) [31], patrinoside aglucone (**6**) [32], patrinoside aglucone-11-*O*-2′-deoxy-*β*-D-glucopyranoside (**7**) [33], and 10-acetylpatrinoside (**8**) [34] (Figure 5) based on the comparison of their spectroscopic data with those in the literature. 

### 2.4. Biological Activities of Compounds ***1***–***8***

All the isolated compounds were assayed for their anti-inflammatory, hepatoprotective, and cytotoxic activities. Among these, compound 3 displayed anti-inflammatory activity by inhibiting the NF-κB pathway, with an inhibitory rate of 73.44% at the concentration of 10 μM, while the other compounds showed weak activity, with an inhibitory rate lower than 50% at 10 μM (Table 2). None of these compounds showed hepatoprotective or cytotoxic activities.

Iridoids are an important type of chemical constituents of the genus *Patrinia*. Previous investigations showed that some iridoids from *P. heterophylla* and *P. scabra* possessed significant anti-inflammatory activity [35,36,37]. However, studies on the iridoids in *P. scabiosifolia* were mainly focused on their cytotoxic and AChE inhibitory activities, and studies on the anti-inflammatory activity of the iridoids from this plant are still insufficient [22,25]. In our work, eight compounds were isolated from *P. scabiosifolia*. Structurally, compounds **1, 3**, and **4** possess the same skeleton, and compound **3** is the aglucone of compound **1**, while compounds **2**, **6**, **7**, and **8** are of the same type, and compound **6** is the aglucone of compound **2**. By comparing the chemical structures of compounds **1**, **3**, and **4** and their anti-inflammatory activities, we found that compound **3,** regarded as the aglucone of compound **1**, had enhanced the anti-inflammatory activity. In addition, when replaced the 4-CH_3_ with a double bond, as in compound **4**, the anti-inflammatory activity dramatically decreased. In contrast, structures such as those of compounds **2**, **6**, **7**, and **8** exhibited no NF-κB inhibition activity. These findings may be helpful for further investigation of structure–activity relationships on this type of compounds.

## 3. Materials and Methods

### 3.1. General

IR data were obtained with a Nicolet is 50 FT-IR spectrometer (Thermo Fisher Scientific, Waltham, MA, USA). Optical rotations were recorded with a JASCO P-2000 spectrometer (JASCO, Easton, MD, USA) at 20 °C. UV and ECD spectra were acquired on JASCO V-650 and JASCO J-815 spectrometers (JASCO, Easton, MD, USA), respectively. HRESIMS data were generated by an Agilent 6520 HPLC-Q-TOF mass spectrometer (Agilent Technologies, Waldbronn, Germany). NMR spectra were obtained with a Bruker AVIII-500 spectrometer (Bruker-Biospin, Billerica, MA, USA), with TMS as an internal standard. A Shimadazu LC-6AD instrument (SPD-20A) (Shimadzu Inc., Kyoto, Japan) was used for preparative HPLC separations. Column chromatography was performed using MCI Gel CHP20P (Mitsubishi Chemical Holdings Corporation; Tokyo, Japan), Sephadex LH-20 (GE Chemical Corporation; Waupaca, WI, USA) and silica gel (Qingdao Marine Chemical Inc.; Qingdao, China); TLC analyses were carried out using GF254 TLC plates (Qingdao Marine Chemical Inc.; Qingdao, China); ODS (50 μm) (Merck; Darmstadt, Germany) was used for MPLC separations. 

### 3.2. Plant Material

The air-dried whole plants of *Patrinia scabiosifolia* Link (20 kg) were collected from Yunnan, China, in June 2018 and identified by Associate Prof. Lin Ma (Institute of Materia Medica, Chinese Academy of Medical Science and Peking Union Medical College). A voucher specimen (ID-24944) was deposited at the herbarium of the Institute of Materia Medica, Chinese Academy of Medical Sciences, and Peking Union Medical College.

### 3.3. Extraction and Isolation

The air-dried plant material (20 kg) of *P. scabiosifolia* was extracted three times with 95% EtOH under reflux for three hours each time and concentrated under reduced pressure to afford a crude residue (3650 g). Part of the residue (2100 g) was extracted by the Soxhlet method sequentially with petroleum ether, dichloromethane, ethyl acetate, and methanol to afford petroleum ether (80 g), CH_2_Cl_2_ (210 g), EtOAc (80 g), and MeOH (1500 g) soluble extracts.

The CH_2_Cl_2_ fraction was applied to a silica gel column and eluted with a gradient of CH_2_Cl_2_–MeOH (100:1–1:1) to give 21 fractions (Fr. A–U). Fr. I was first applied to a medium-pressure MCI column (MeOH–H_2_O 4:6) and then purified by Sephadex LH-20 (CH_2_Cl_2_–MeOH 1:1) to give compound **5** (1.5 mg). Fr. N was chromatographed on a silica gel column (CH_2_Cl_2_−MeOH, 100:1–1:1) to obtain 5 fractions (Fr. N.1–5). Fr. N.3 was then separated by a Sephadex LH-20 column (CH_2_Cl_2_–MeOH 1:1) and further purified by semipreparative HPLC (18% MeOH, *v* = 2 mL/min) to give compounds **3** (183.4 mg, t_R_ = 28 min) and **4** (64.8 mg, t_R_ = 25 min). Fr. Q was subjected to silica gel chromatography (CH_2_Cl_2_−MeOH, 50:1–1:1) to afford 5 subfractions (Fr. Q.1–5). Fr. Q.3 was then separated by Sephadex LH-20 chromatography (MeOH–H_2_O 4:6) and purified by semipreparative HPLC (20% acetonitrile, *v* = 2 mL/min) to yield compound **6** (2.1 mg, t_R_ = 36 min).

The EtOAc portion was chromatographed on an MCI column, eluting with a gradient mixture of MeOH–H_2_O (3:7–10:0) to give 6 fractions (Fr. A–F). Fr. C (13.0 g) was subjected to silica gel chromatography (CH_2_Cl_2_−MeOH, 80:1–1:1) to obtain 9 fractions (Fr. C1–C9). Fr. C7 (3.3 g) was separated by medium-pressure ODS to afford 8 subfractions (Fr. C7a–C7h). Fr. C7d was subsequently subjected to Sephadex LH-20 chromatography (MeOH–H_2_O 1:1) and further purified by semipreparative HPLC (40% MeOH, *v* = 2 mL/min) to give compounds **2** (16.2 mg, t_R_ = 34 min) and **7** (2.2 mg, t_R_ = 37 min); Fr. C7e was fractionated and purified using the same procedure described above, leading to the isolation of compounds **1** (19.8 mg, t_R_ = 26 min) and **8** (12.6 mg, t_R_ = 28 min).

### 3.4. Spectroscopic Data of Compounds ***1*** and ***2***

Patriniscabioside A (**1**): white powder; [α${]}_{D}^{20}$+29 (*c* 0.10, MeOH); UV (MeOH): *λ*_max_ (log *ε*) 205 nm (2.46); IR *v*_max_ 3383, 2960, 2930, 2875, 1733, 1458, 1398, 1369, 1334, 1294, 1239, 1190, 1169, 1081, 1050, 1024, 961, 895, 616 cm^−1^; ^1^H and ^13^C NMR data, see Table 1; HRESIMS *m/z* 447.22247 [M + H]^+^ (calcd. C_21_H_35_O_10_, 447.22247). 

Patriniscabioside B (**2**): light yellow oil; [α${]}_{D}^{20}$-58 (*c* 1.0, MeOH); UV (MeOH): *λ*_max_ (log *ε*) 204nm (4.11); IR *v*_max_ 3377, 2957, 2932. 2874, 1747, 1667, 1466, 1412, 1370, 1292, 1251, 1150, 1117, 1088, 1067, 1037, 1008, 983, 934, 889, 617 cm^−1^; ^1^H and ^13^C NMR data, see Table 1; HRESIMS *m/z* 491.21338 [M + HCOO]^−^ (calcd. C_22_H_35_O_12_, 491.21340). 

### 3.5. Anti-Inflammatory Assay for Compounds ***1***–***8***

The anti-inflammatory activity of all the isolated compounds was assayed in 293T cells transiently transfected with the pNF-κB-Luc expression plasmid. The pRL-CMV-Renilla plasmid was co-transfected as a control. After transfection, the cells were pretreated with the test compounds for 1 h and then stimulated with lipopolysaccharide (LPS, 1 μg/mL) for 24 h. A luciferase assay was performed with the Dual-Luciferase Reporter Assay System following the manufacturer’s instructions (Promega, Madison, WI, USA). The assay was conducted according to a previous published paper [38]. Compounds **1**–**8** were dissolved in DMSO at a concentration of 10 μM. JSH23 [4-methyl-*N*^1^-(3-phenylpropyl)-benzene-1,2-diamine] was used as positive control.

## 4. Conclusions

As a result, two new iridoid glucosides, patriniscabiosides A (**1**) and B (**2**), together with six known iridoids and iridoid glucosides (**3**~**8**), were isolated from the whole plants of *Patrinia scabiosifolia*, and their structures were elucidated by various spectroscopic analyses including 1D and 2D NMR, IR, UV, and HRESIMS. The absolute configuration of the new compounds was further confirmed by ECD. Among these, compound **1** is an iridoid glucoside with an isovaleryl moiety attached to the 6′-position of the glucose, and compound **2** possesses a rare 4-deoxyglucose moiety. In addition, compounds **4, 7, 8** are reported from *P. scabiosifolia* for the first time. Compound **3** displayed potent anti-inflammatory activity through the inhibition of the NF-κB pathway, with an inhibitory rate of 73.44% at a concentration of 10 μM. The cytotoxic and hepatoprotective activities of compounds **1**–**8** were also tested, but were not relevant. According to our bioassay results, we propose that the iridoid lactone skeleton, as well as the methyl group at C-4 can contribute to the anti-inflammatory activity, while its glucoside would, somehow, weaken its bioactivity. We believe our findings will provide important information for researchers in future work; further studies on the anti-inflammatory mechanism of the isolated compound are still needed.

## Figures and Tables

**Table 1:** ^1^H-NMR (500 MHz) and ^13^C-NMR (125 MHz) spectroscopic data of compounds **1** and **2** (in CD_3_OD, *δ* in ppm, *J* in Hz).

Compounds	1		2	
Position	*δ*_C_	*δ*_H_	*δ*_C_	*δ*_H_
**1**	178.1		93.6	5.91, d, *J* = 5.4 Hz
**2**				
**3**	74.4	4.14, dd, *J* = 10.7, 3.2 Hz 3.97, t, *J* = 10.7 Hz	140.0	6.37, s
**4**	37.3	1.57, m	116.5	
**5**	43.3	2.39, m	34.1	3.03, q-like, *J* = 7.7 Hz
**6**	40.5	2.38, m 1.33, m	40.9	2.07, m 1.83, ddd, *J* = 13.1, 8.0, 4.9 Hz
**7**	83.7	4.26, br.t, *J* = 3.8 Hz	73.3	4.33, td, *J* = 4.9, 3.0 Hz
**8**	51.3	2.44, m	49.1	1.96, m
**9**	42.7	2.81, m	42.7	2.18, m
**10**	61.8	3.89, dd, *J* = 11.0, 4.8 Hz 3.82, dd, *J* = 11.0, 9.0 Hz	62.2	3.82, dd, *J* = 10.9, 7.5 Hz 3.73, dd, *J* = 10.9, 5.7 Hz
**11**	15.8	0.99, d, *J* = 6.7 Hz	69.6	4.25, d, *J* = 11.5 Hz 4.07, d, *J* = 11.5 Hz
**saccharide moiety**				
**1′**	105.2	4.39, d, *J* = 7.8 Hz	103.8	4.23, d, *J* = 7.8 Hz
**2′**	75.3	3.13, dd, *J* = 9.1, 7.8 Hz	76.9	3.10, dd, *J* = 8.9, 7.8 Hz
**3′**	77.9	3.34, t, *J* = 9.1 Hz	72.2	3.60, m
**4′**	71.5	3.26, dd, *J* = 9.7, 9.1 Hz	36.4	1.92, m 1.36, m
**5′**	75.4	3.44, ddd, *J* = 9.7, 5.8, 2.1 Hz	73.9	3.53, m
**6′**	64.3	4.47, dd, *J* = 11.8, 2.1 Hz 4.14, dd, *J* = 11.8, 5.8 Hz	65.6	3.57, m
**isovaleryl moiety**				
**1″**	174.5		172.3	
**2″**	44.3	2.24, d, *J* = 6.8 Hz 2.25, d, *J* = 7.5 Hz	44.1	2.24, d, *J* = 6.8 Hz 2.25, d, *J* = 7.5 Hz
**3″**	27.0	2.06, m	26.8	2.09, m
**4″**	22.8	0.95, d, *J* = 6.7 Hz	22.6	0.97, d, *J* = 6.8 Hz
**5″**	22.8	0.95, d, *J* = 6.7 Hz	22.6	0.97, d, *J* = 6.8 Hz

**Table 2:** NF-κB-Dependent Reporter Gene Expression Assay results for compounds **1**–**8**.

Compounds	Concentration	Inhibitory Rate * (%)
JSH-23	10 μM	78.92
1	10 μM	48.39
2	10 μM	0.00
3	10 μM	73.44
4	10 μM	48.39
5	10 μM	13.55
6	10 μM	0.00
7	10 μM	0.00
8	10 μM	6.88

* Compounds with inhibitory rate lower than 50% were considered inactive.

## Data Availability

The data presented in this study are available in supplementary material.

## Supplementary Materials

The following are available online. UV, IR, HRESIMS, CD, 1D- and 2D- NMR spectra for compounds **1** and **2**, ^1^H and ^13^C NMR spectra and data for compounds **3**~**8**, as well as optimized conformers of (1*S*,5*S*,7*S*,8*S*,9*S*)-**2** and (1*R*,5*R*,7*R*,8*R*,9*R*)-**2**.

## Sample Availability

Samples of the compounds **1–8** are available from the authors.
