# Supplementary material for: Two New Iridoid Glucosides from the Whole Plant of Patrinia scabiosifolia Link
Source: Molecules. 2021 Jul 10;26(14):4201. doi: 10.3390/molecules26144201 (PMC8304010; doi:10.3390/molecules26144201)
Supplement: Supplementary file 1 [file molecules-26-04201-s001.zip › molecules-1250575-supplementary.pdf]

Supplementary Materials:

Two new iridoid glucosides from the whole plants of *Patrinia scabiosifolia* Link

Shi-Kai Sun†, Jiang Fu†, Kai-Dong Liu, Ming-Zhu Dai, Yong Li, Yun-Bao Liu, Shuang-Gang Ma, Jing Qu*

Figure S1. IR spectrum of compound **1**.

Figure S2. UV spectrum of compound **1**.

Figure S3. HRESIMS spectrum of compound **1**.

Figure S4. ^{1}H NMR spectrum of compound **1**.

Figure S5. ^{13}C NMR spectrum of compound **1**.

Figure S6. ^{1}H-^{1}H COSY spectrum of compound **1**.

Figure S7. HSQC spectrum of compound **1**.

Figure S8. HMBC spectrum of compound **1**.

Figure S9. ROESY spectrum of compound **1**.

Figure S10. Experimental ECD spectrum of compound **1**.

Figure S11. IR spectrum of compound **2**.

Figure S12. UV spectrum of compound **2**.

Figure S13. HRESIMS spectrum of compound **2**.

Figure S14. ^{1}H NMR spectrum of compound **2**.

Figure S15. ^{13}C NMR spectrum of compound **2**.

Figure S16. ^{1}H-^{1}H COSY spectrum of compound **2**.

Figure S17. HSQC spectrum of compound **2**.

Figure S18. HMBC spectrum of compound **2**.

Figure S19. ROESY spectrum of compound **2**.

Figure S20. Experimental ECD spectrum of compound **2**.

Figure S21. ^{1}H NMR spectrum of compound **3**.

Figure S22. ^{13}C NMR spectrum of compound **3**.

Figure S23. ^{1}H NMR spectrum of compound **4**.

Figure S24. ^{13}C NMR spectrum of compound **4**.

Figure S25. ^{1}H NMR spectrum of compound **5**.

Figure S26. ^{13}C NMR spectrum of compound **5**.

Figure S27. ^{1}H NMR spectrum of compound **6**.

Figure S28. ^{13}C NMR spectrum of compound **6**.

Figure S29. ^{1}H NMR spectrum of compound **7**.

Figure S30. ^{13}C NMR spectrum of compound **7**.

Figure S31. ^{1}H NMR spectrum of compound **8**.

Figure S32. ^{13}C NMR spectrum of compound **8**.

Table S1. B3LYP/6-31g(d) optimized low-energy conformers for (1*S*,5*S*,7*S*,8*S*,9*S*)-**2**.

Table S2. B3LYP/6-31g(d) optimized low-energy conformers for (1*R*,5*R*,7*R*,8*R*,9*R*)-**2**.

Table S3. ^{1}H NMR and ^{13}C NMR data of compounds **3~5**.

Table S4. ^{1}H NMR and ^{13}C NMR data of compounds **6~8**.

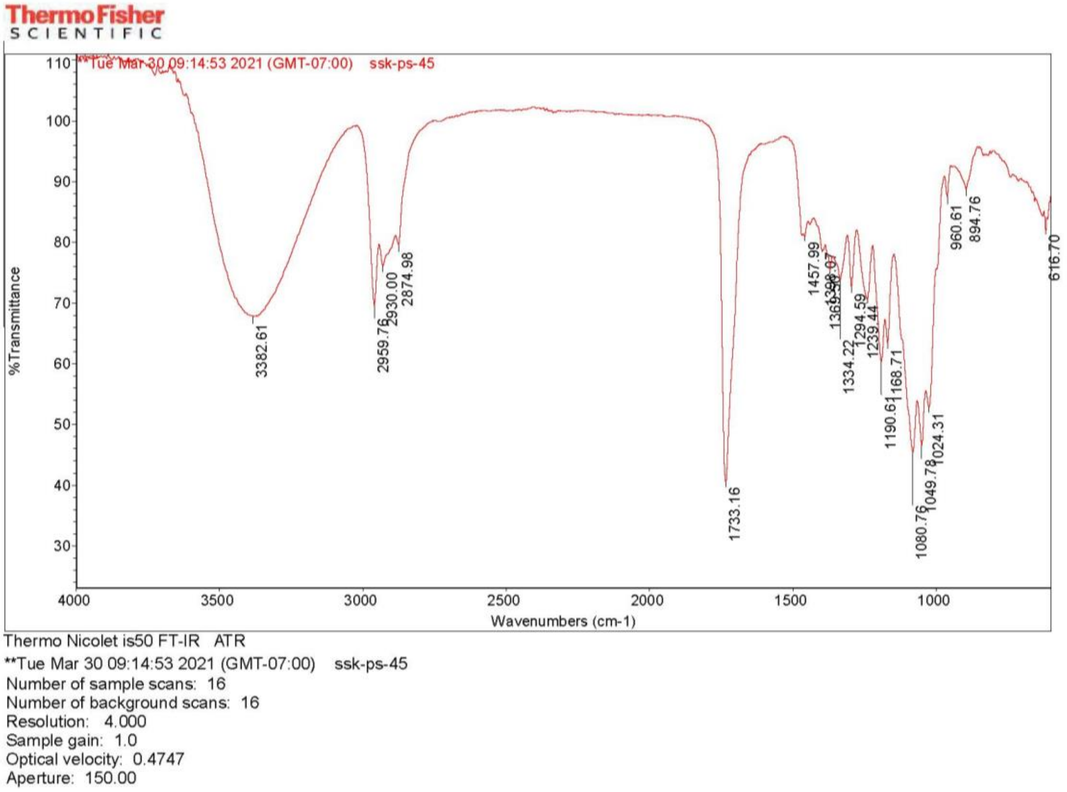

Figure S1. IR spectrum of compound **1**.

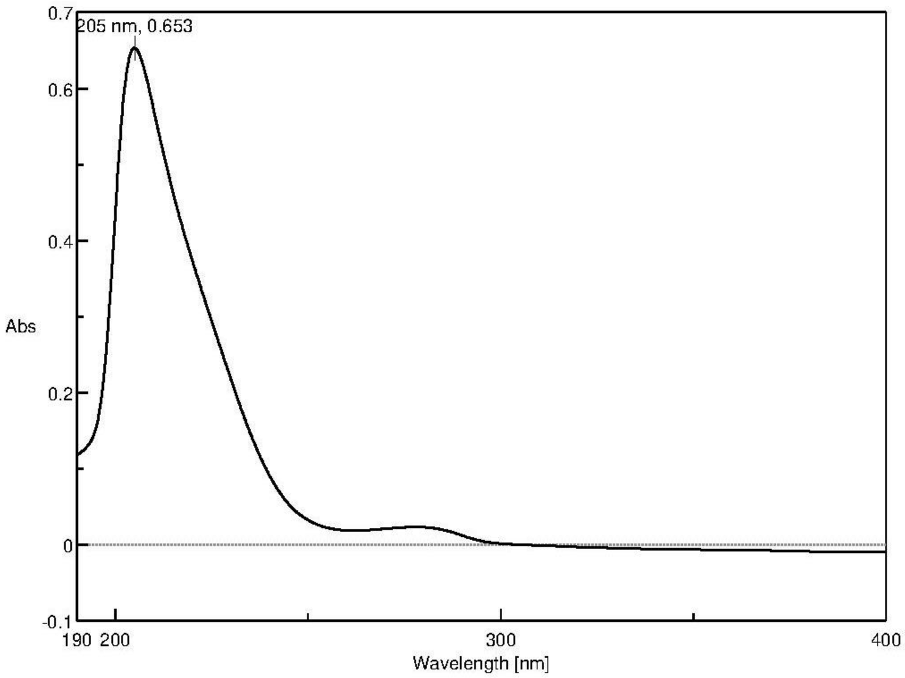

Figure S2. UV spectrum of compound **1**.

Thermo Qexactive Focus Report

compound NO. : SSK-PS-45
Method : LCMS(compound)-low

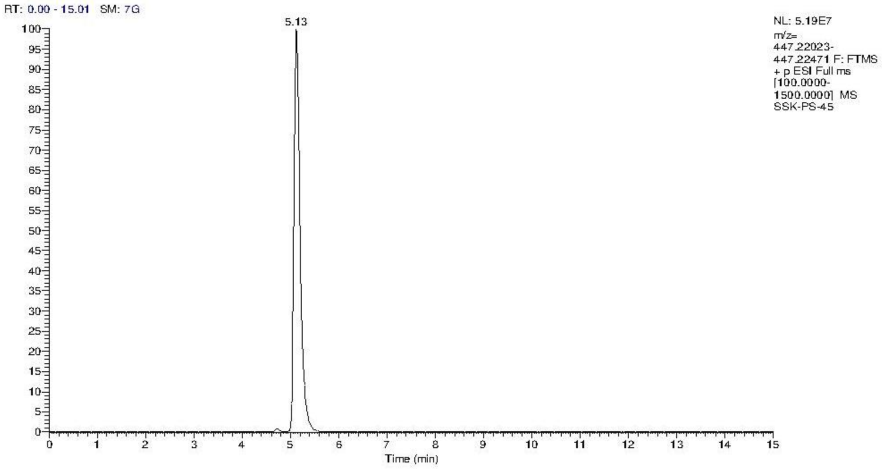

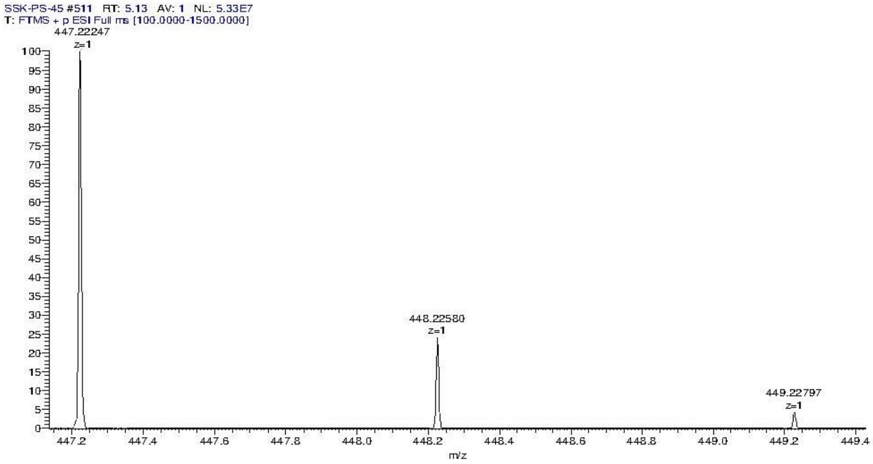

m/z	Theo. Mass	Delta (ppm)	RDB equiv.	Composition	
447.22247	447.22247	-0.01	4.5	C21 H35 O10	M+H

Figure S3. HR-ESI-MS spectrum of compound **1**.

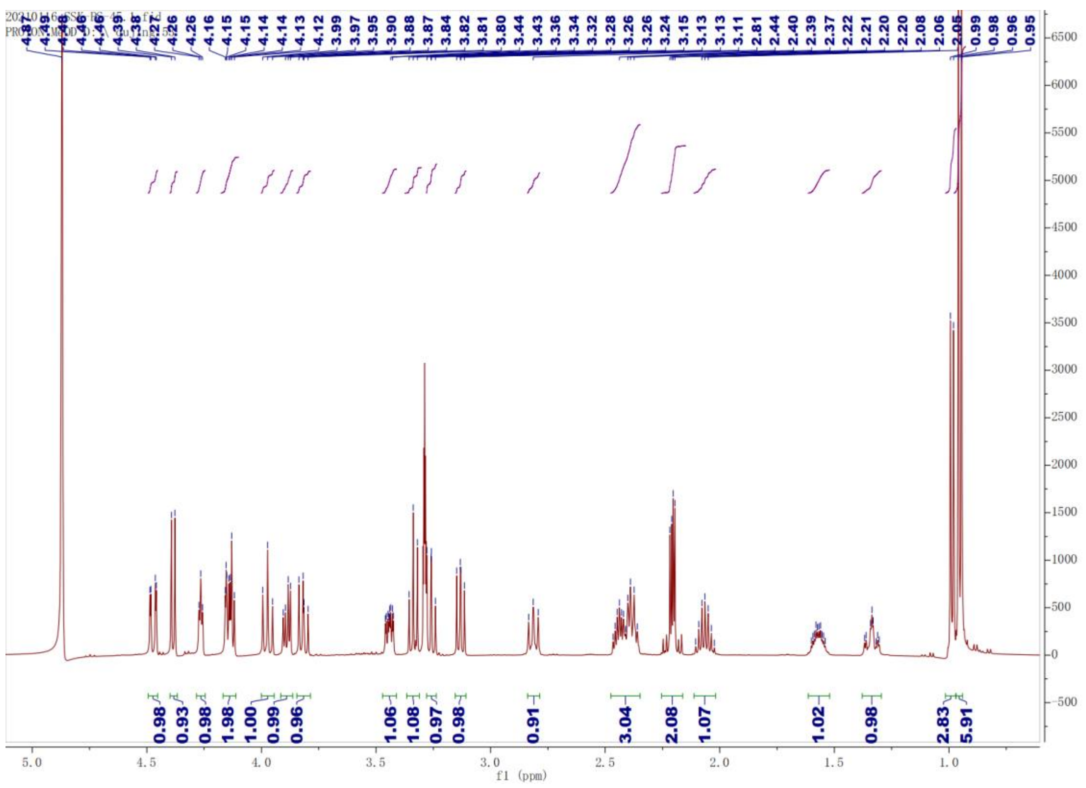

Figure S4. ^{1}H NMR spectrum of compound **1**.

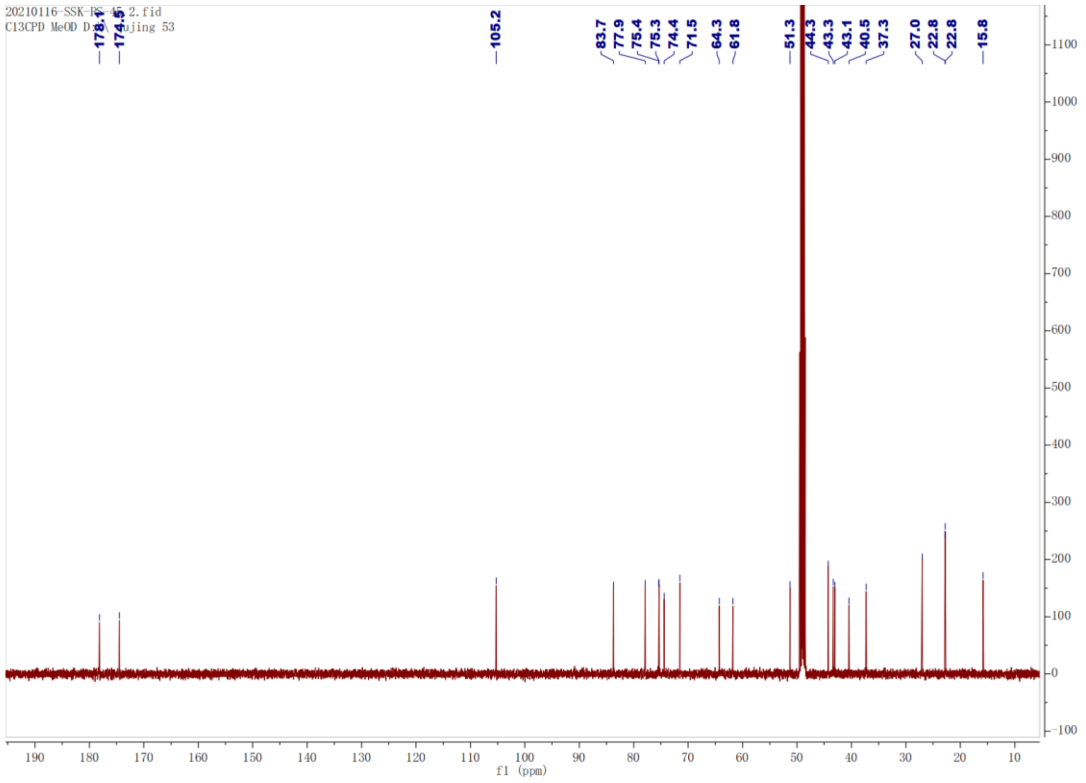

Figure S5. ^{13}C NMR spectrum of compound **1**.

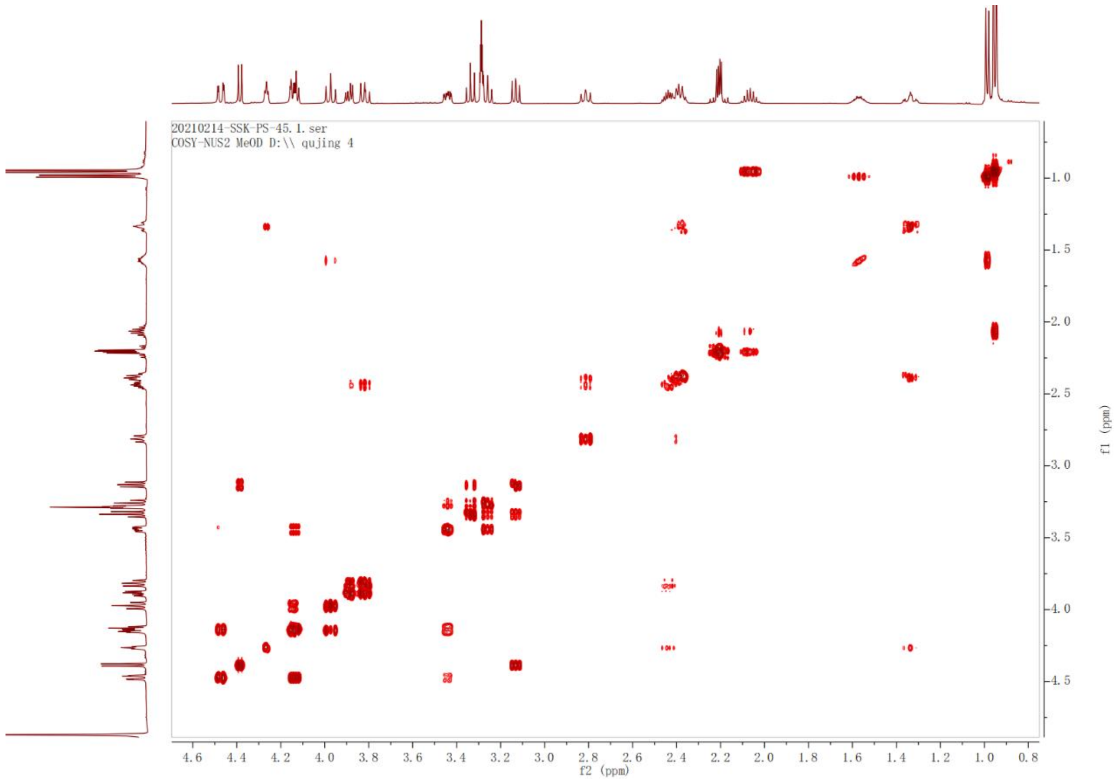

Figure S6. ^{1}H-^{1}H COSY spectrum of compound **1**.

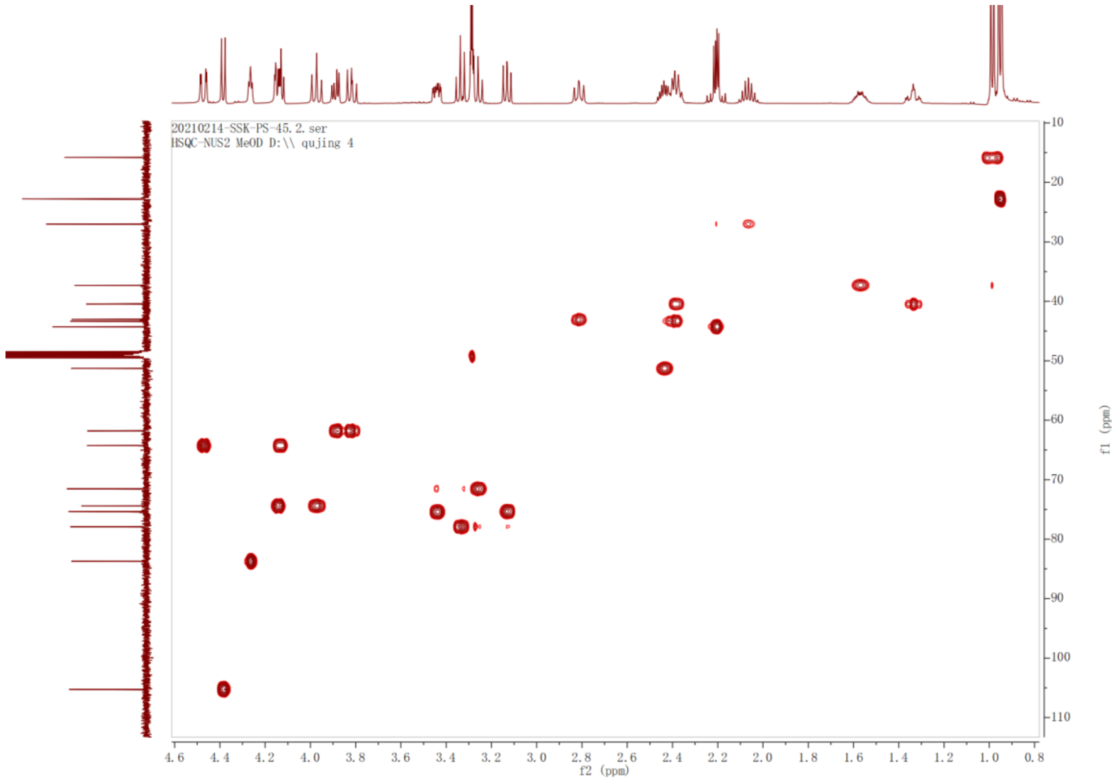

Figure S7. HSQC spectrum of compound **1**.

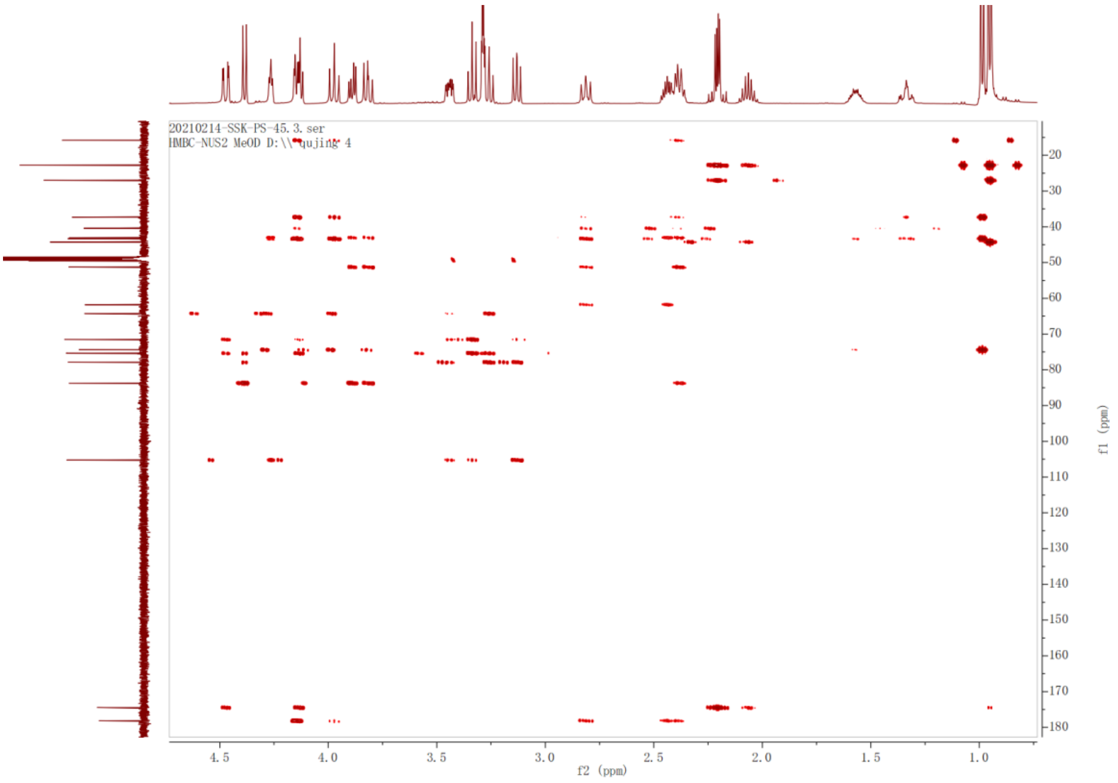

Figure S8. HMBC spectrum of compound **1**.

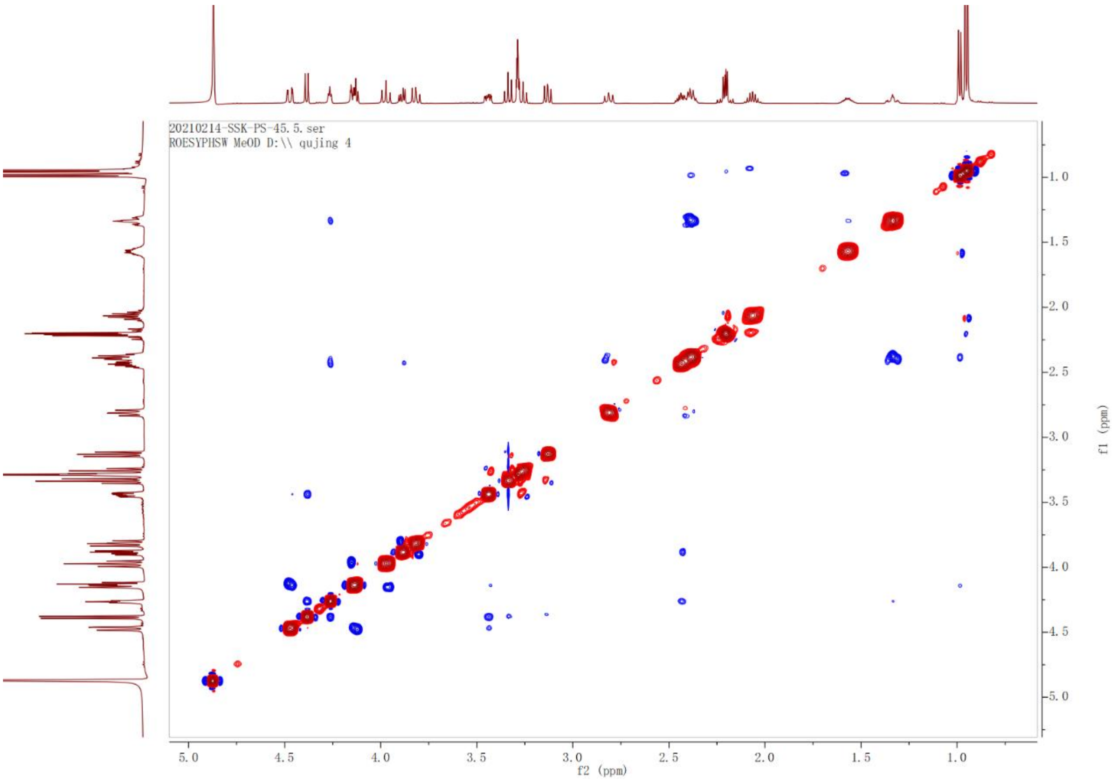

Figure S9. ROESY spectrum of compound **1**.

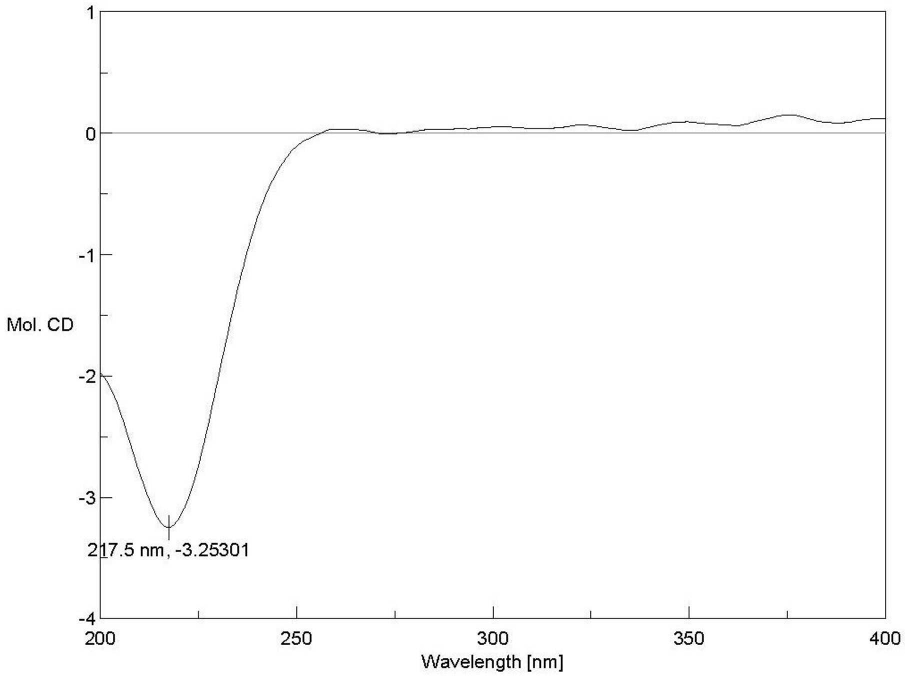

Figure S10. Experimental ECD spectrum of compound **1**.

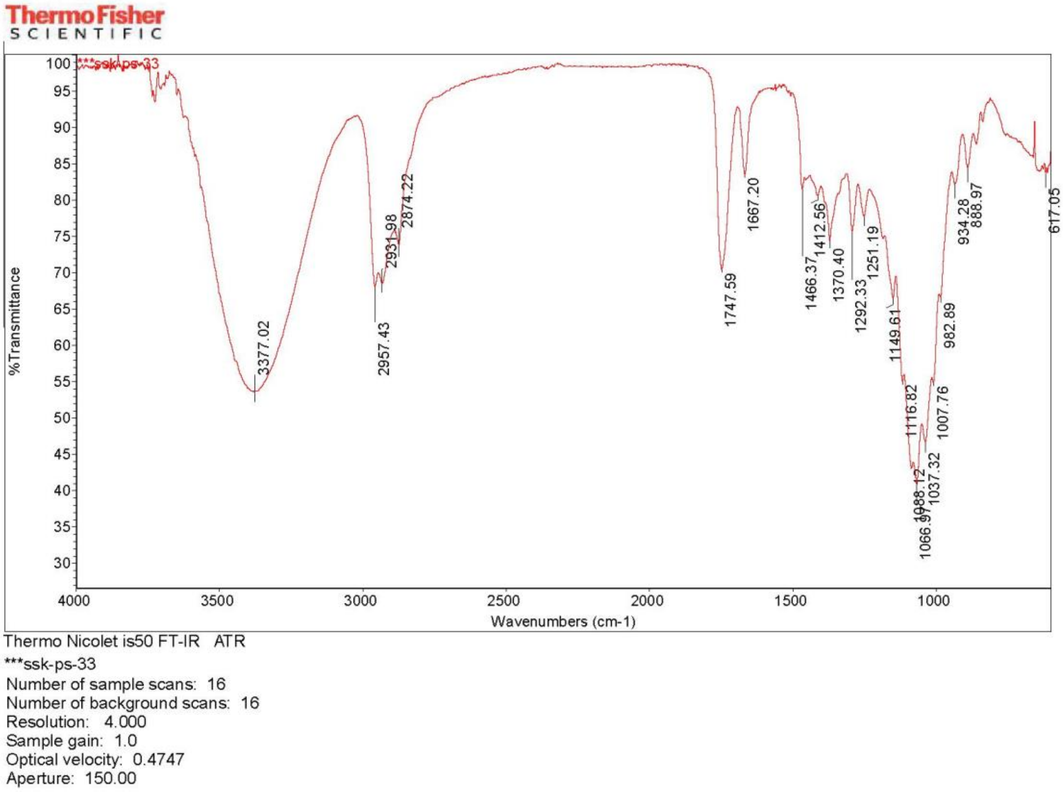

Figure S11. IR spectrum of compound **2**.

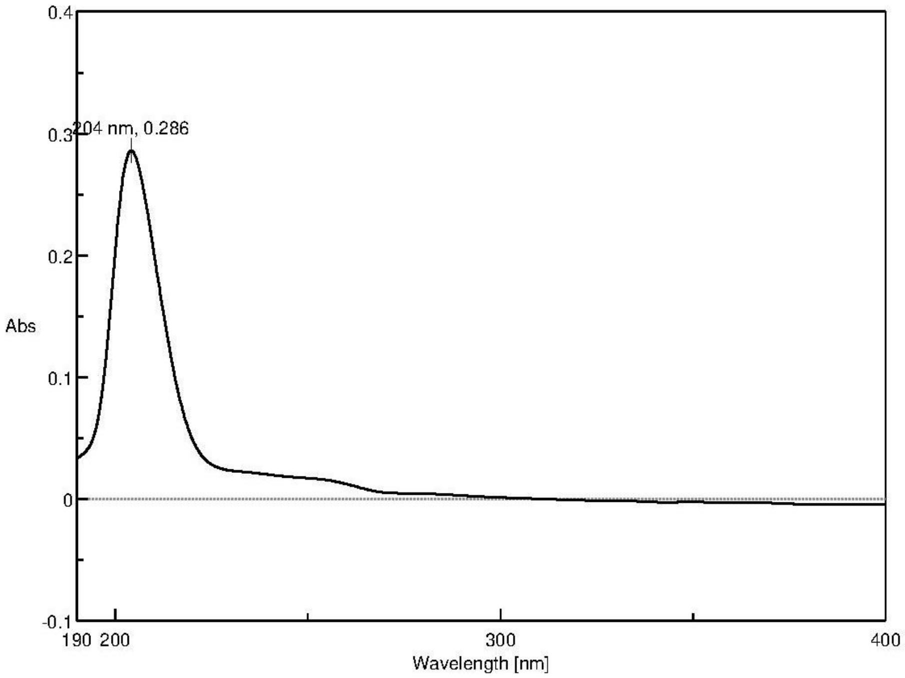

Figure S12. UV spectrum of compound **2**.

Thermo Qexactive Focus Report

compound NO. : SSK-PS-33
Method : LCMS(compound)-low

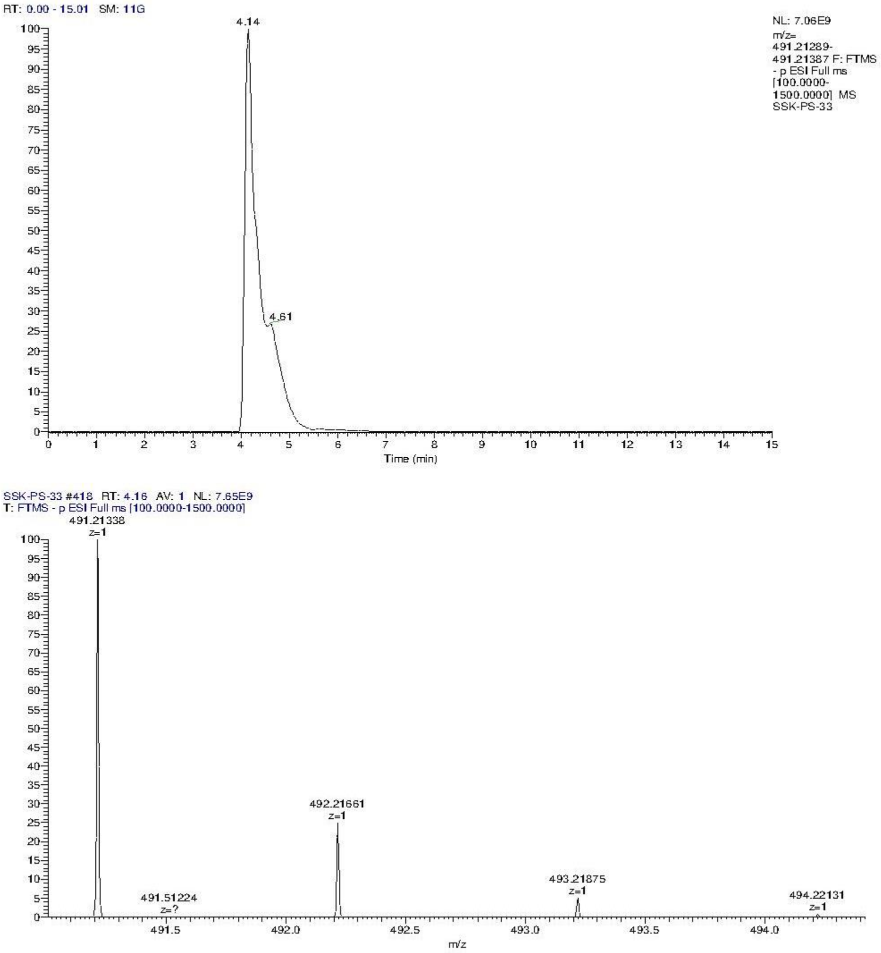

m/z	Theo. Mass	Delta (ppm)	RDB equiv.	Composition	
491.21338	491.21340	-0.04	5.5	C22 H35 O12	M+HCOO

Figure S13. HR-ESI-MS spectrum of compound **2**.

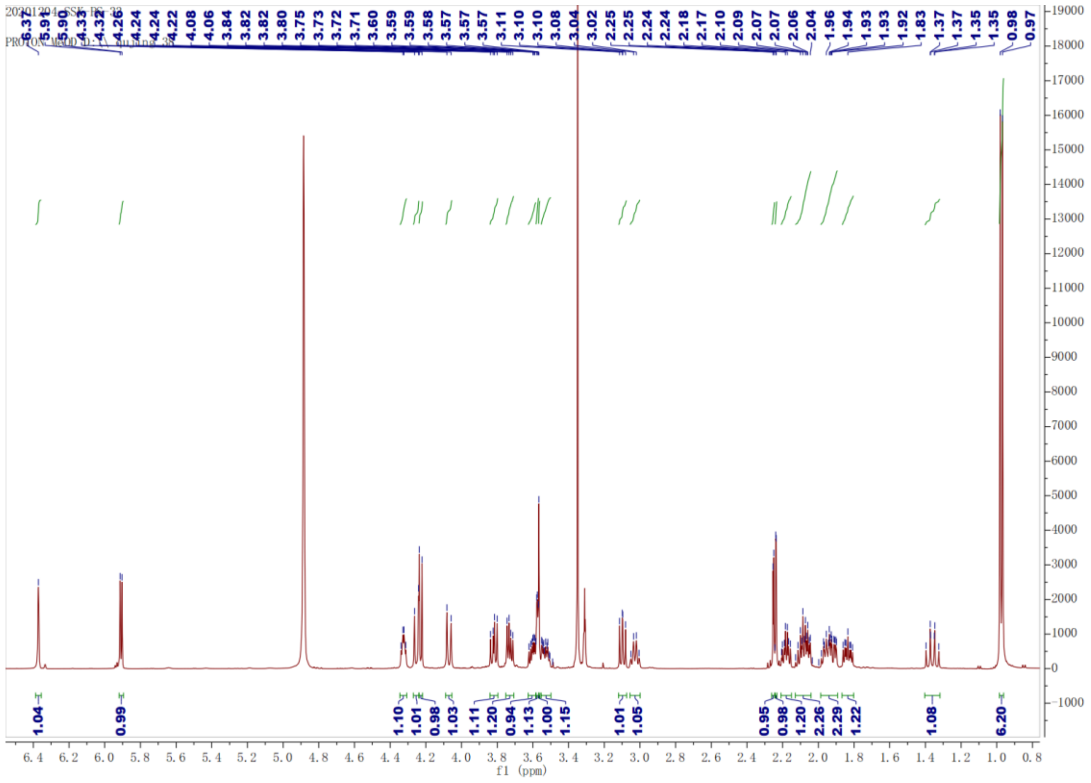

Figure S14. ^{1}H NMR spectrum of compound **2**.

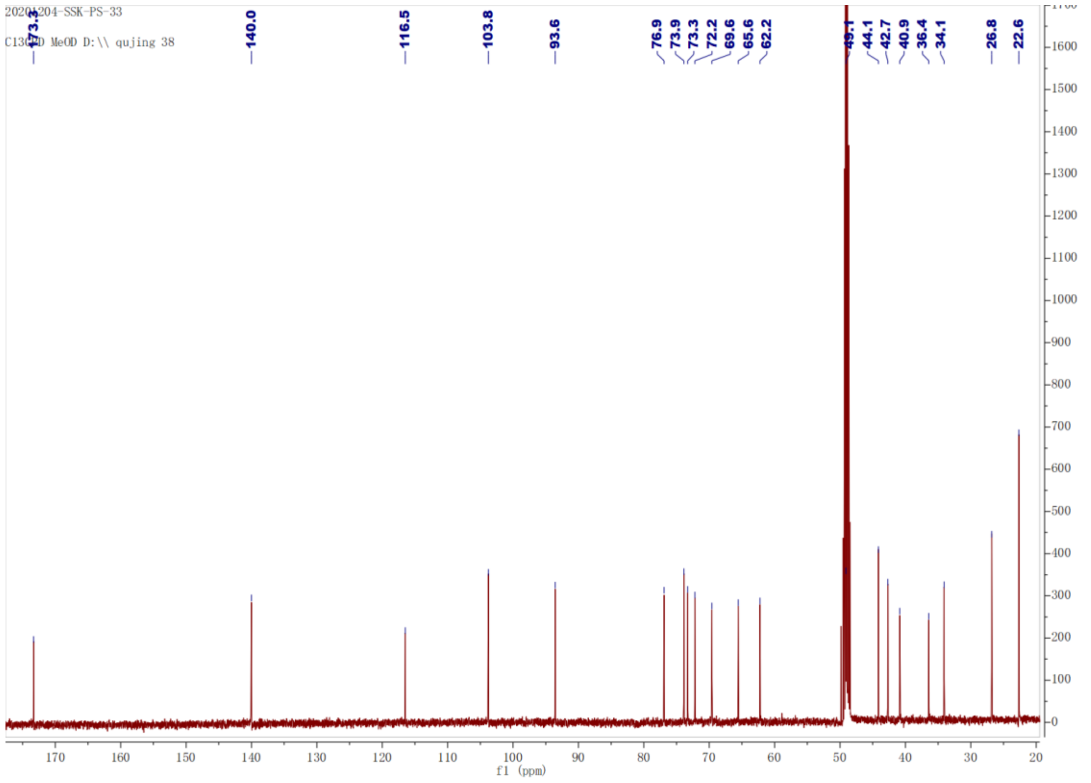

Figure S15. ^{13}C NMR spectrum of compound **2**.

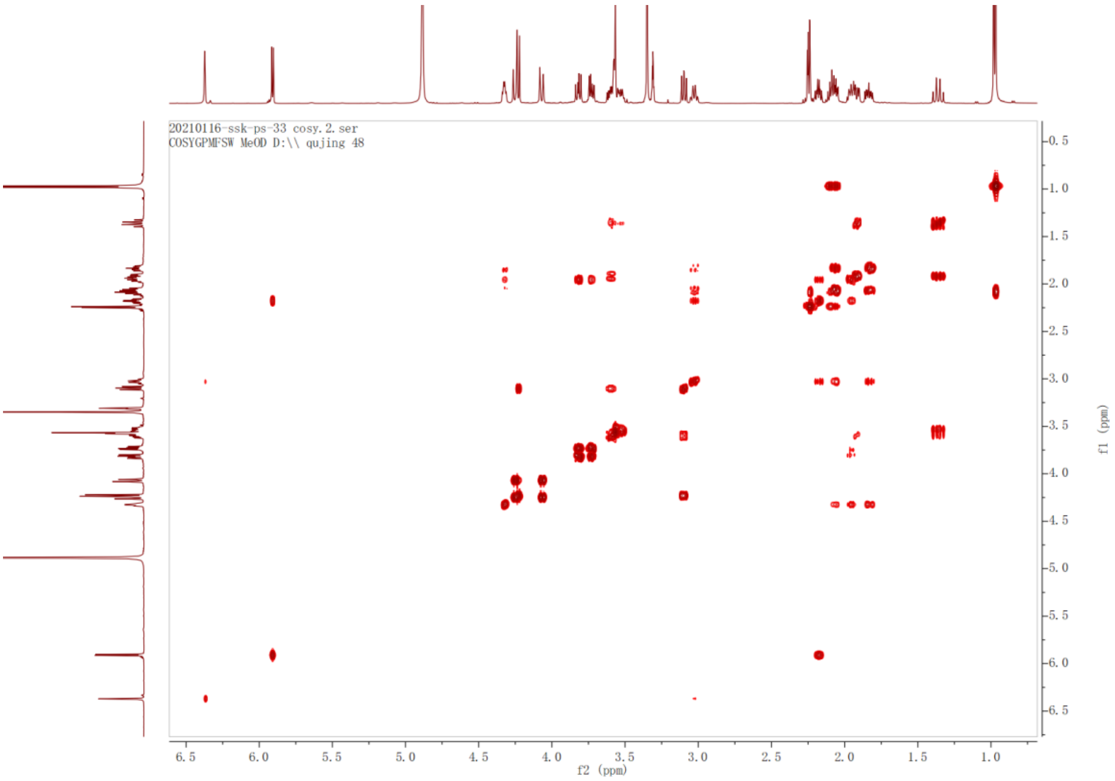

Figure S16. ^{1}H-^{1}H COSY spectrum of compound **2**.

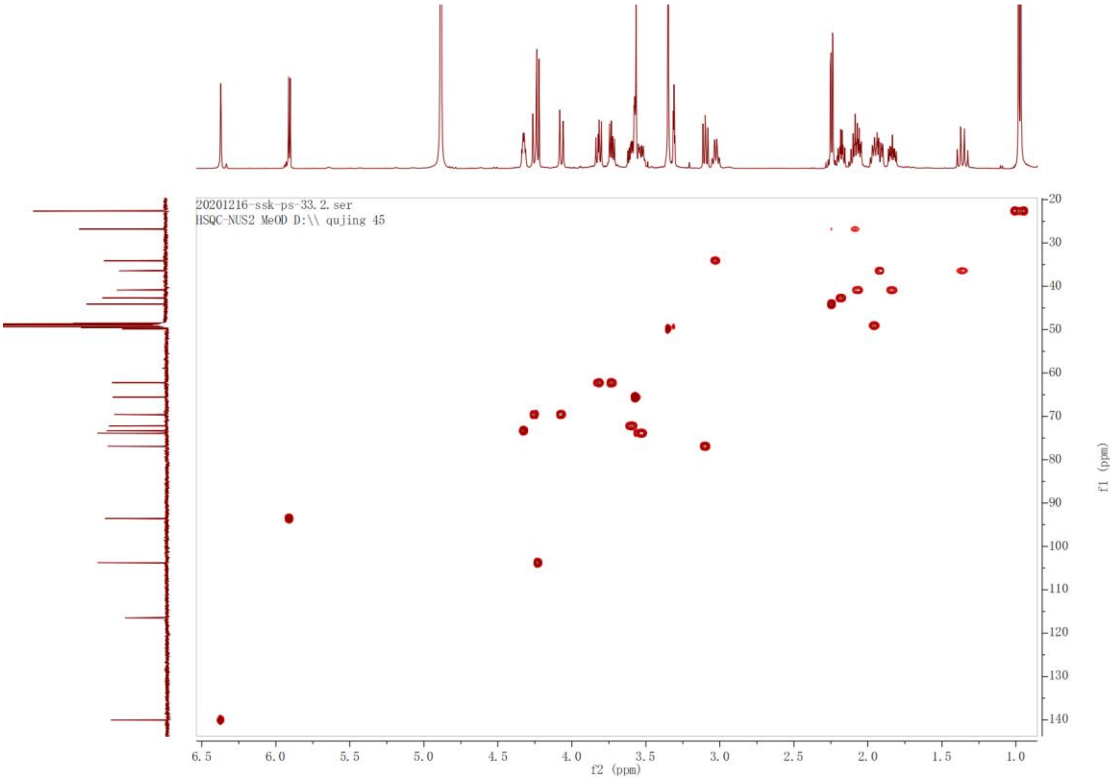

Figure S17. HSQC spectrum of compound **2**.

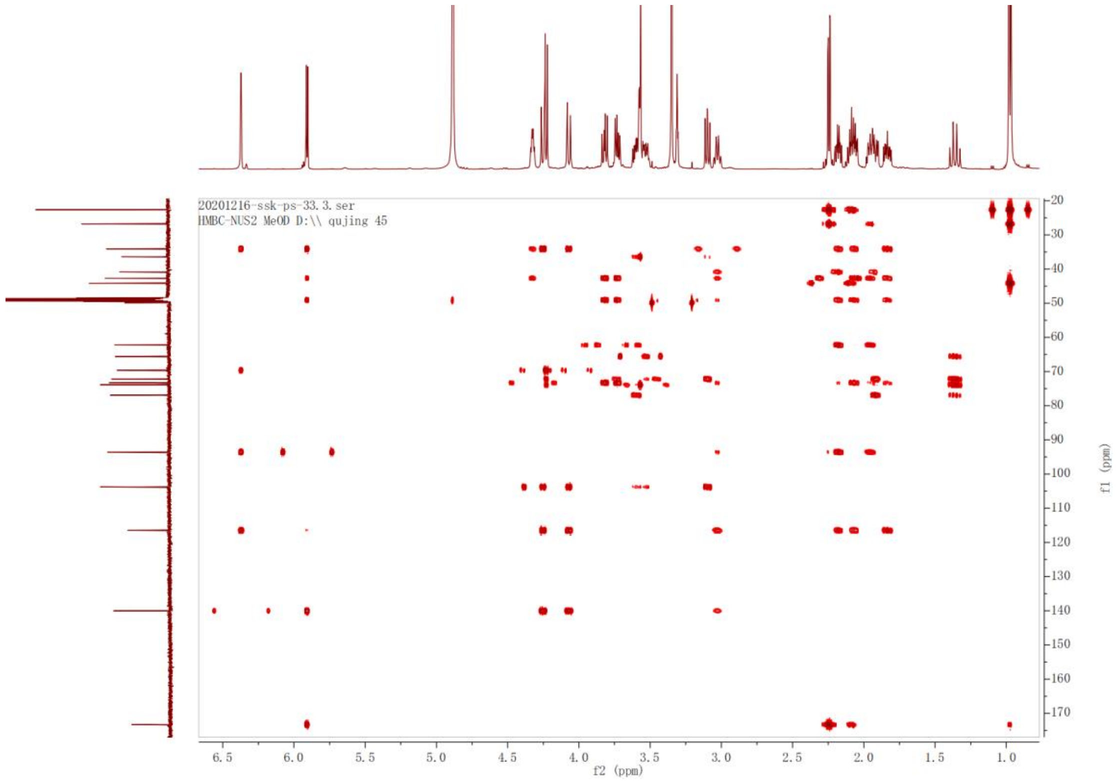

Figure S18. HMBC spectrum of compound **2**.

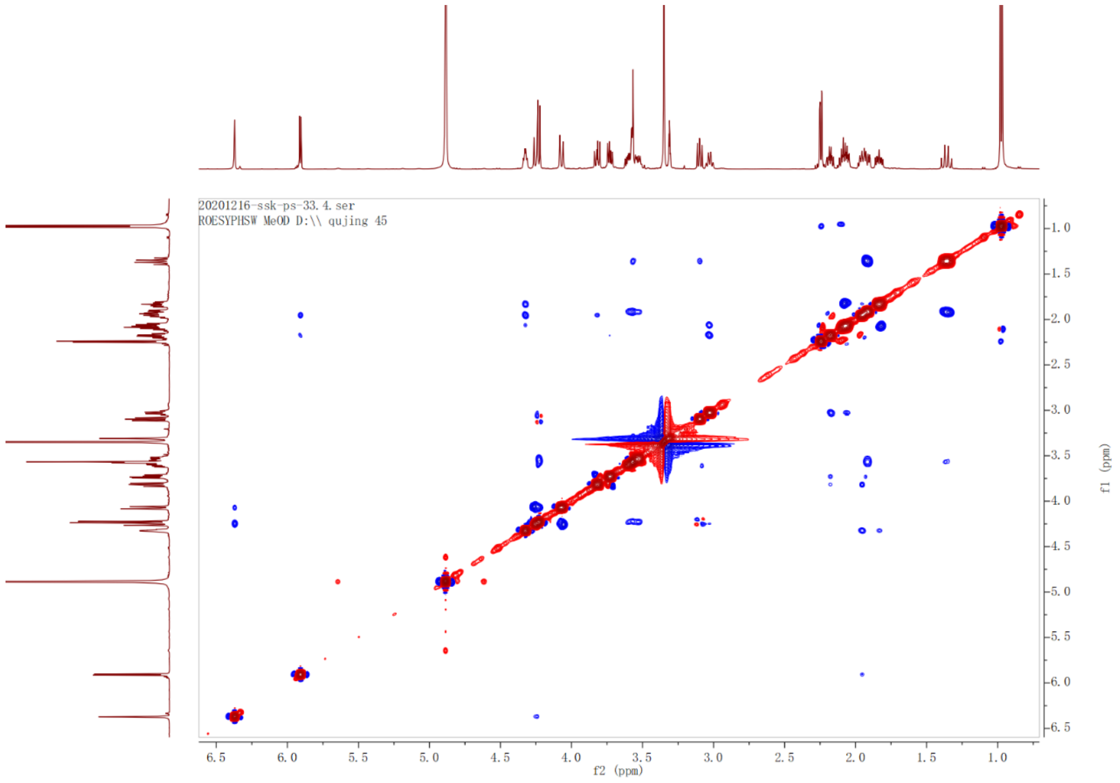

Figure S19. ROESY spectrum of compound **2**.

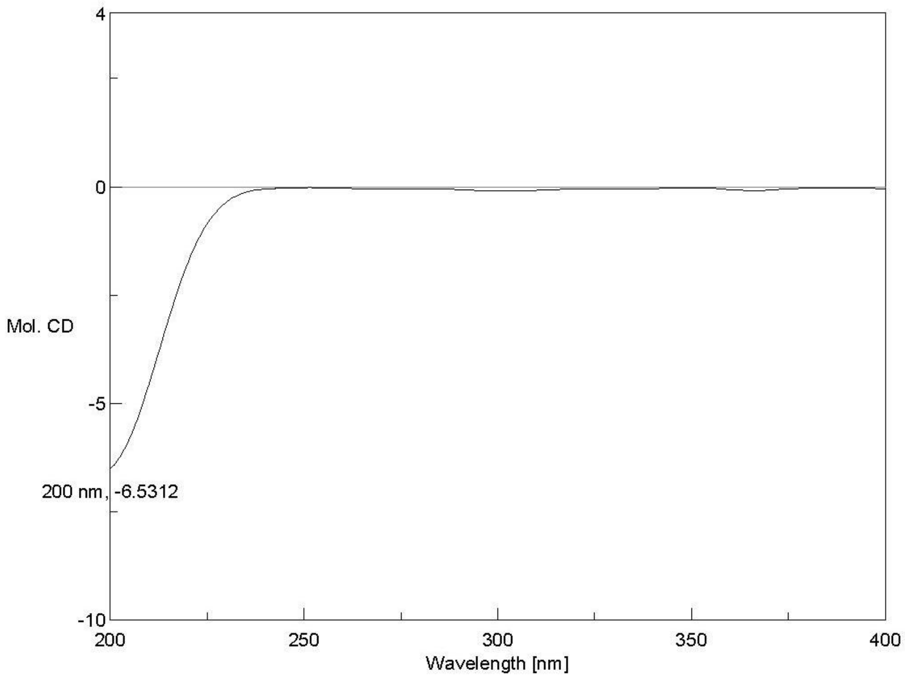

Figure S20. Experimental ECD spectrum of compound **2**.

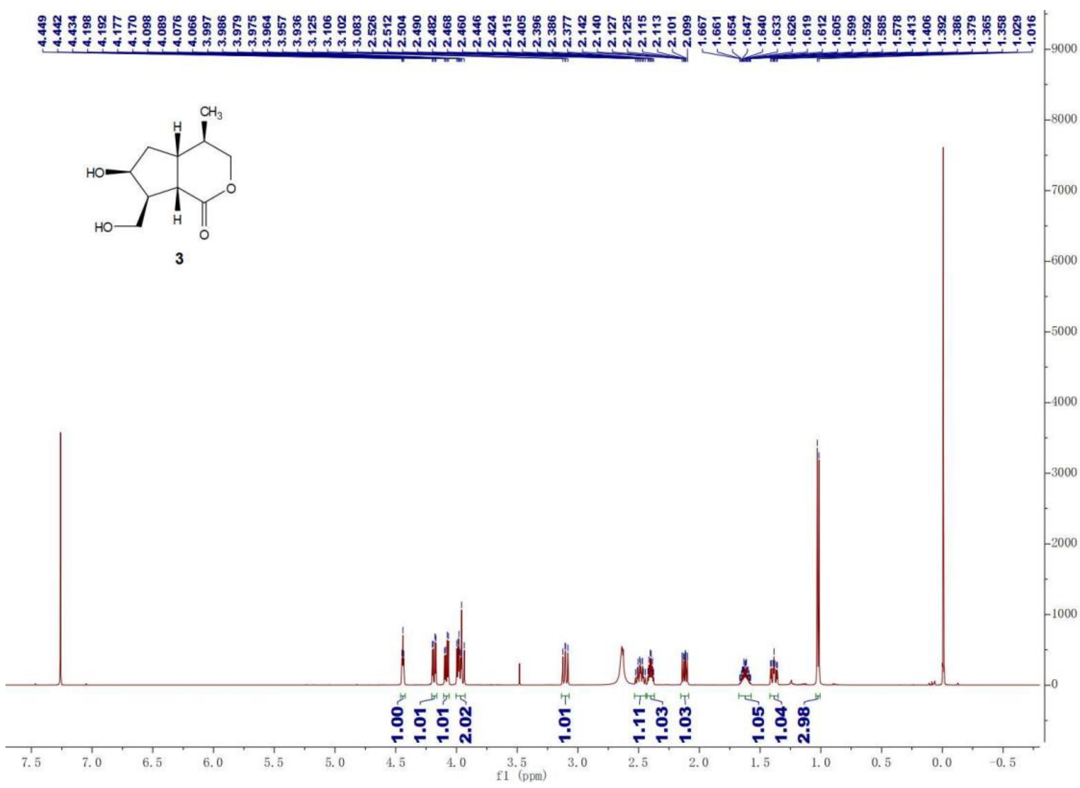

Figure S21. ^{1}H NMR spectrum of compound **3**.

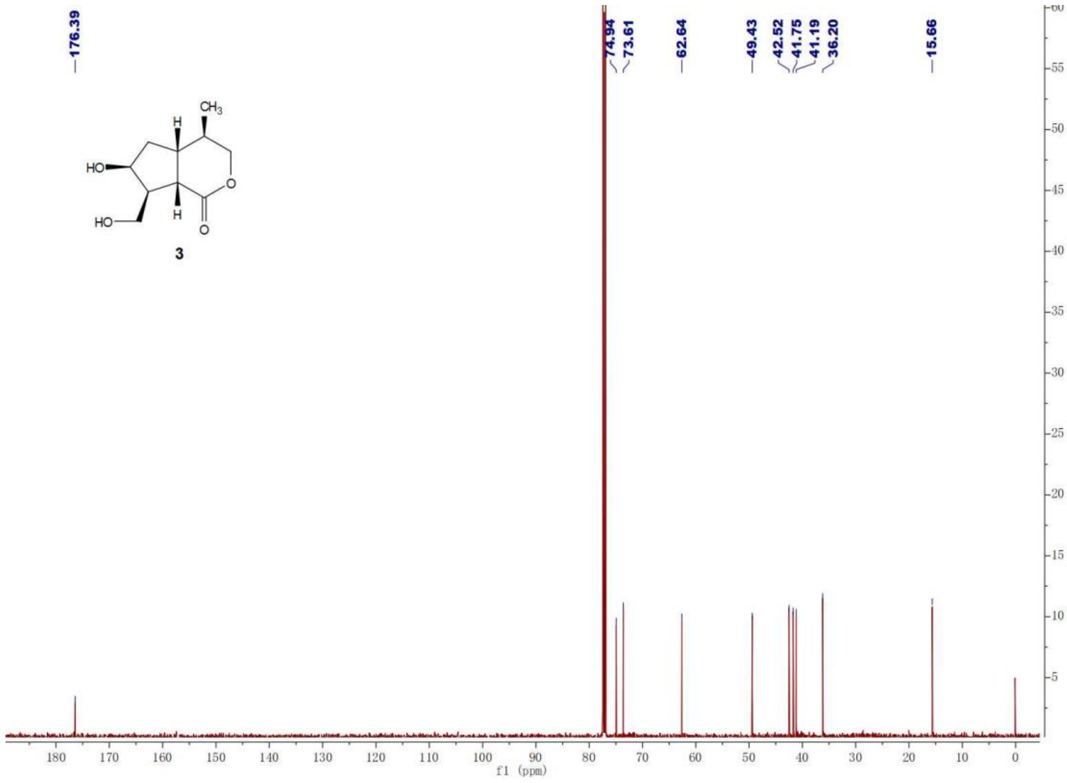

Figure S22. ^{13}C NMR spectrum of compound **3**.

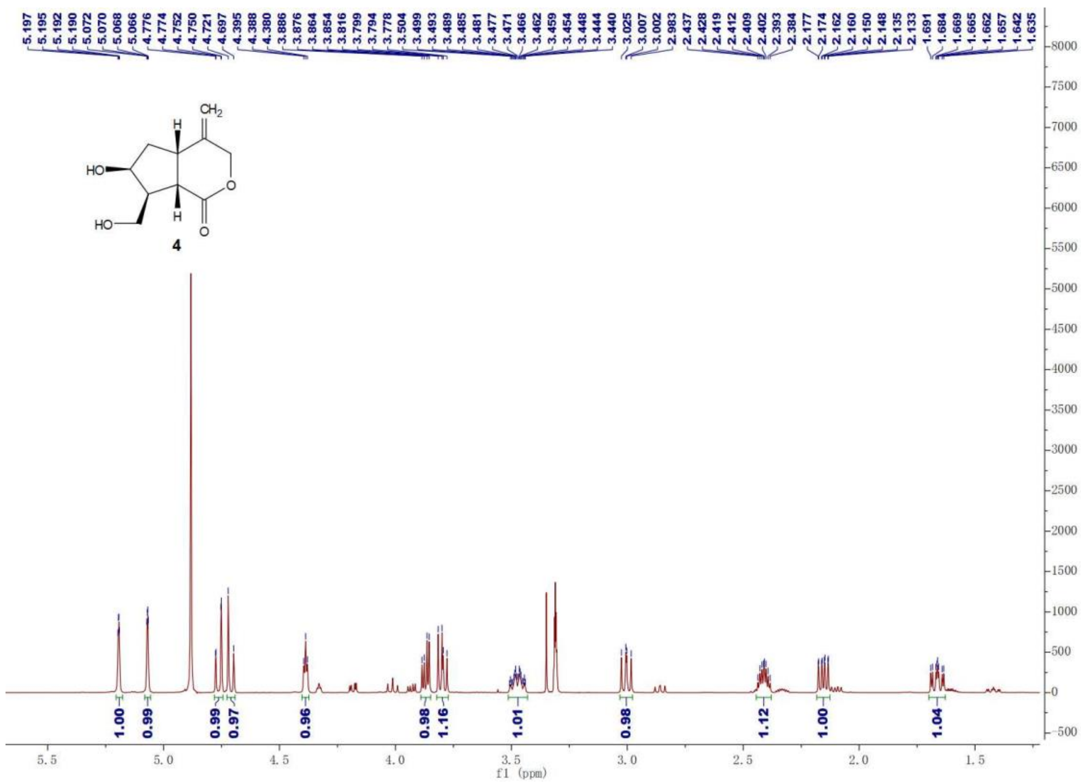

Figure S23. ^{1}H NMR spectrum of compound **4**.

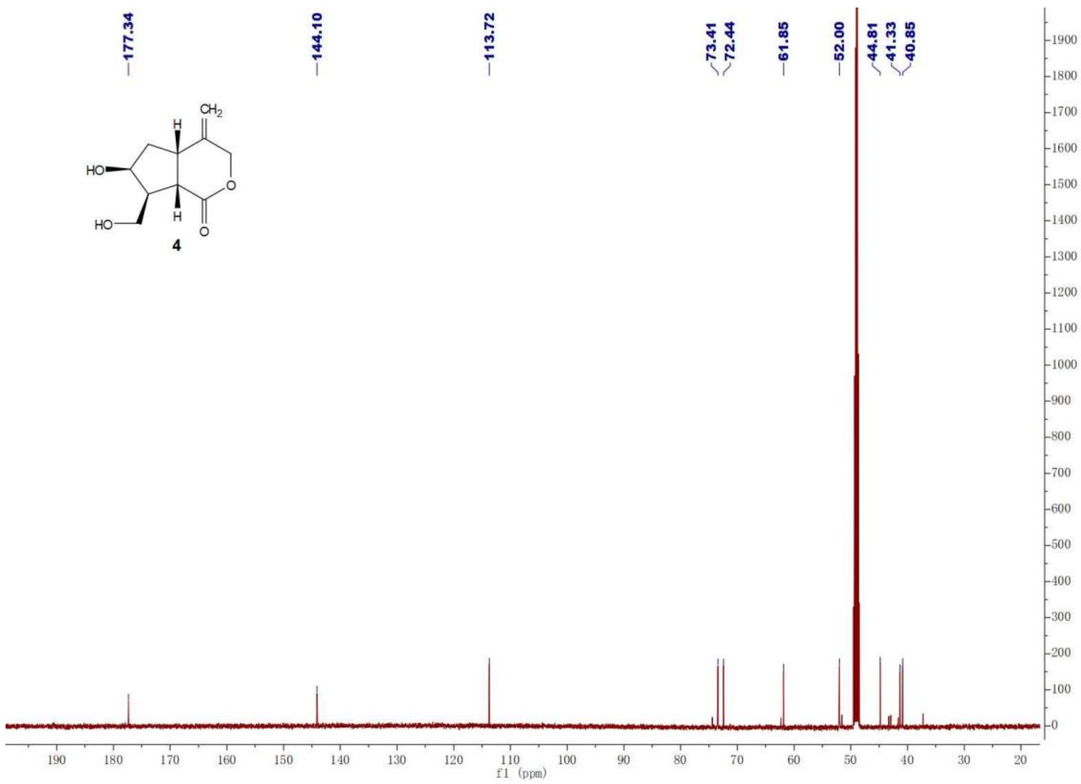

Figure S24. ^{13}C NMR spectrum of compound **4**.

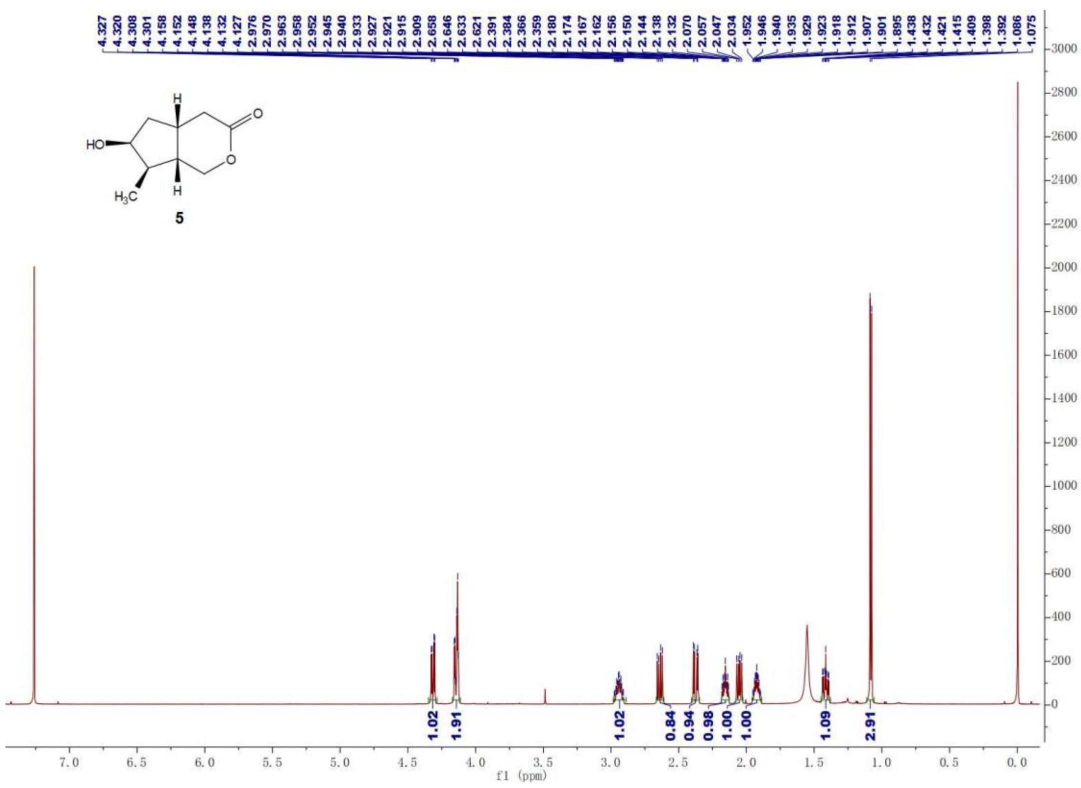

Figure S25. ^{1}H NMR spectrum of compound **5**.

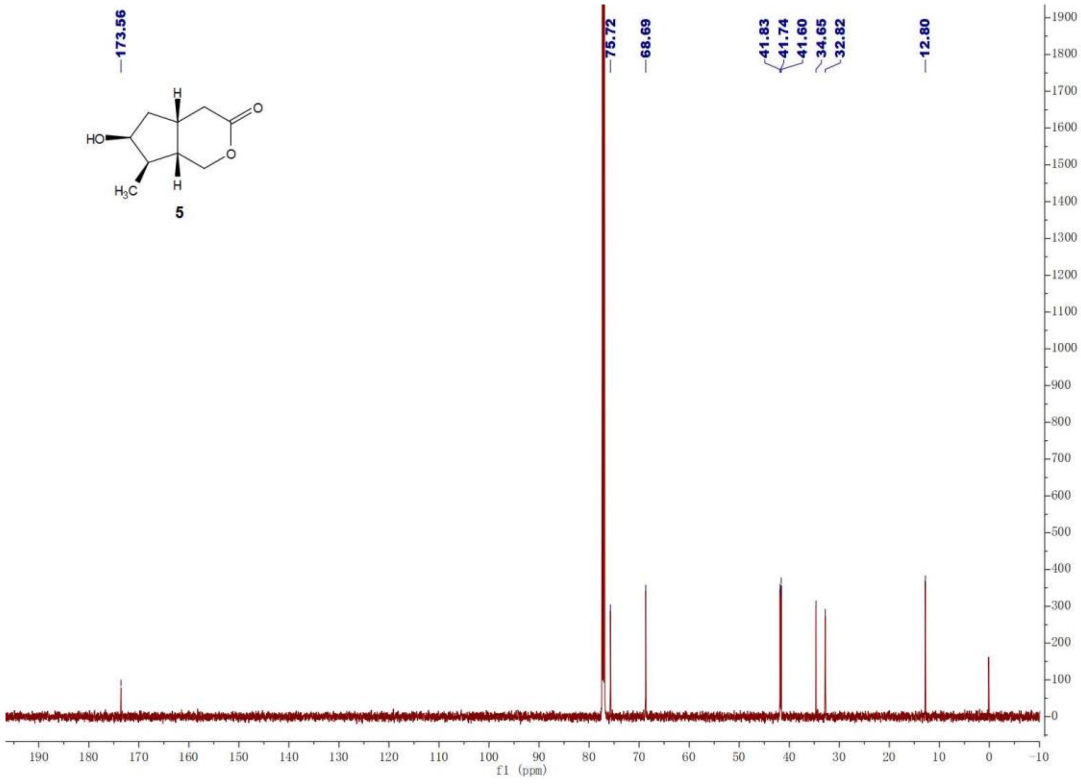

Figure S26. ^{13}C NMR spectrum of compound **5**.

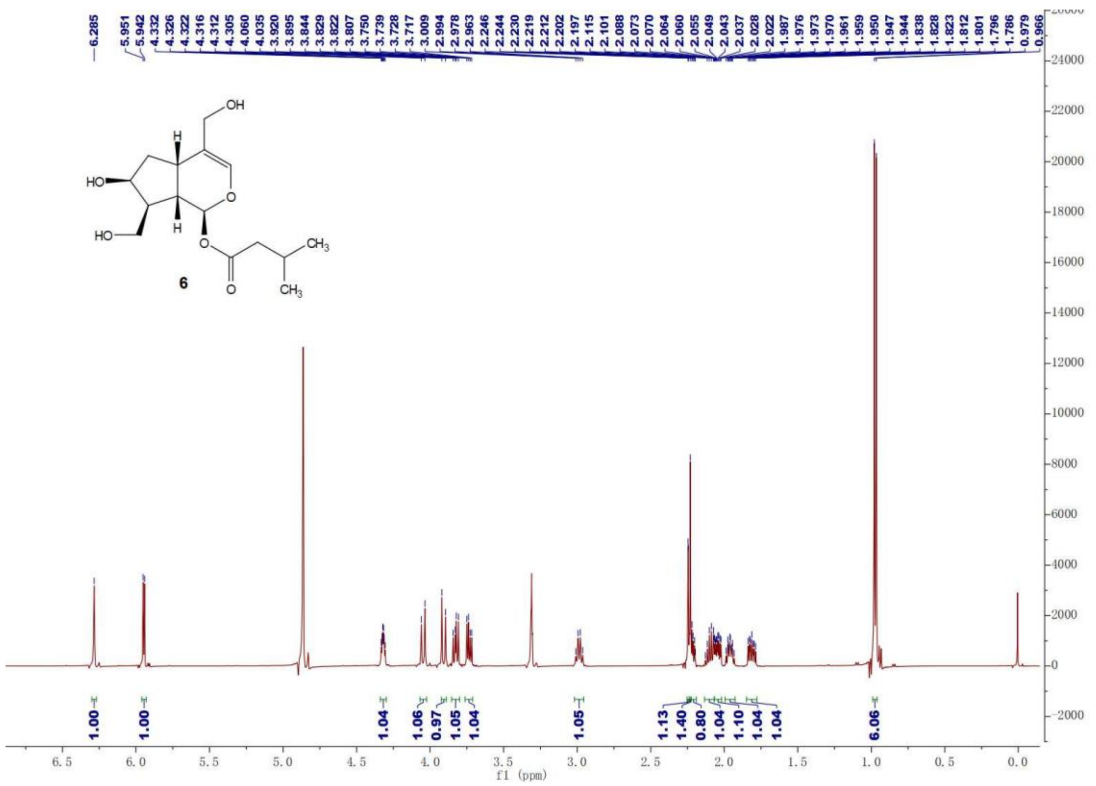

Figure S27. ^{1}H NMR spectrum of compound **6**.

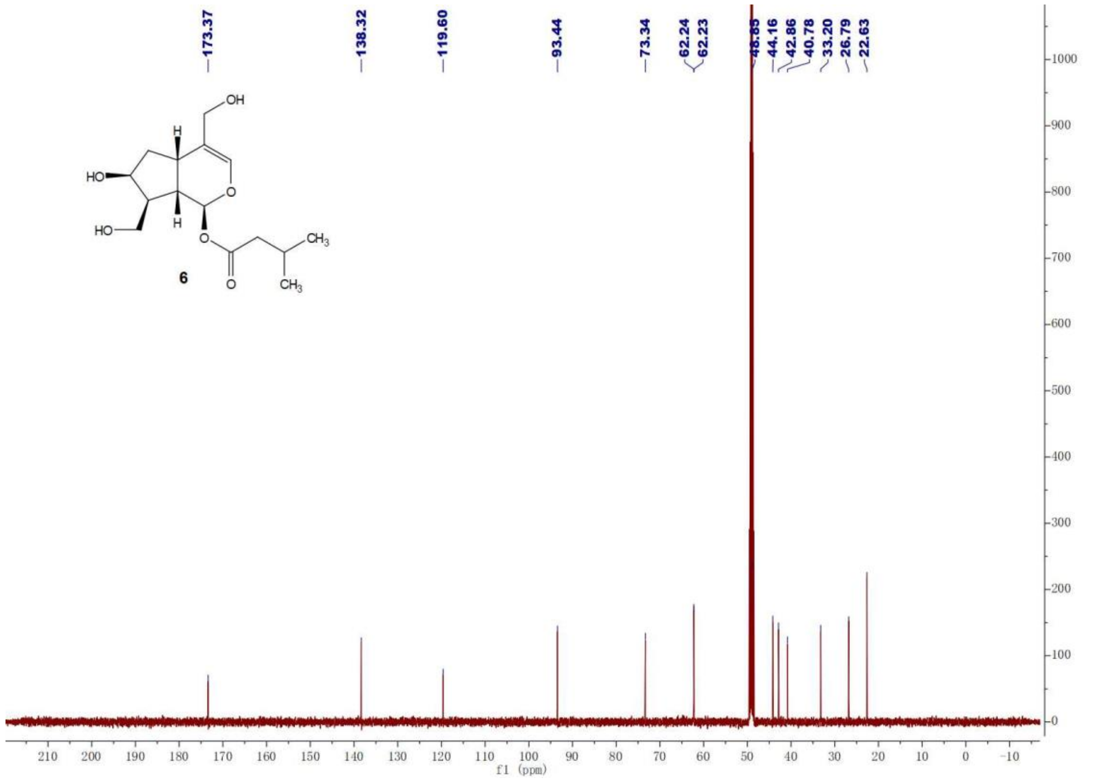

Figure S28. ^{13}C NMR spectrum of compound **6**.

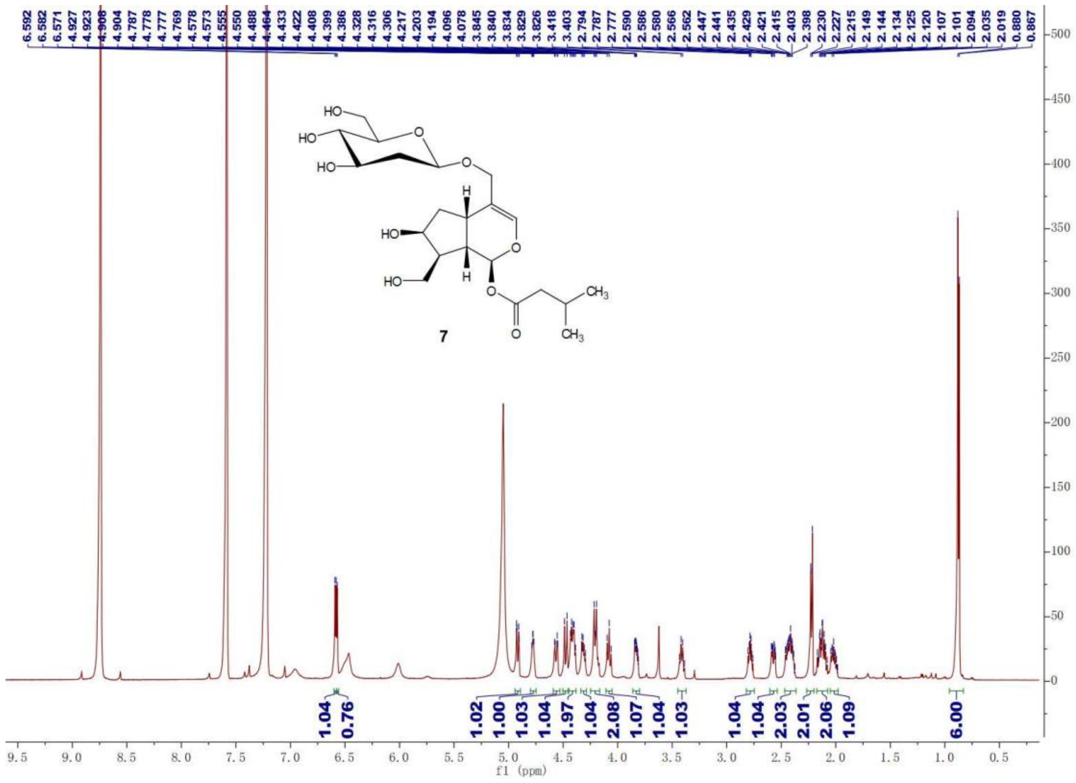

Figure S29. ^{1}H NMR spectrum of compound **7**.

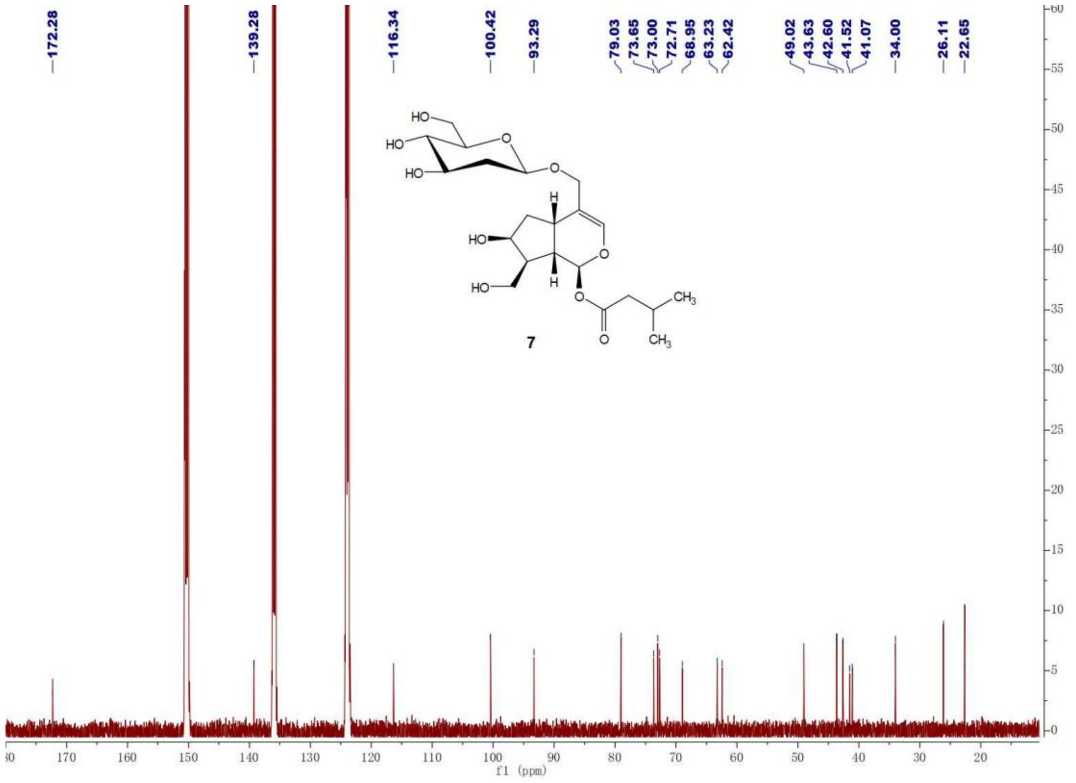

Figure S30. ^{13}C NMR spectrum of compound **7**.

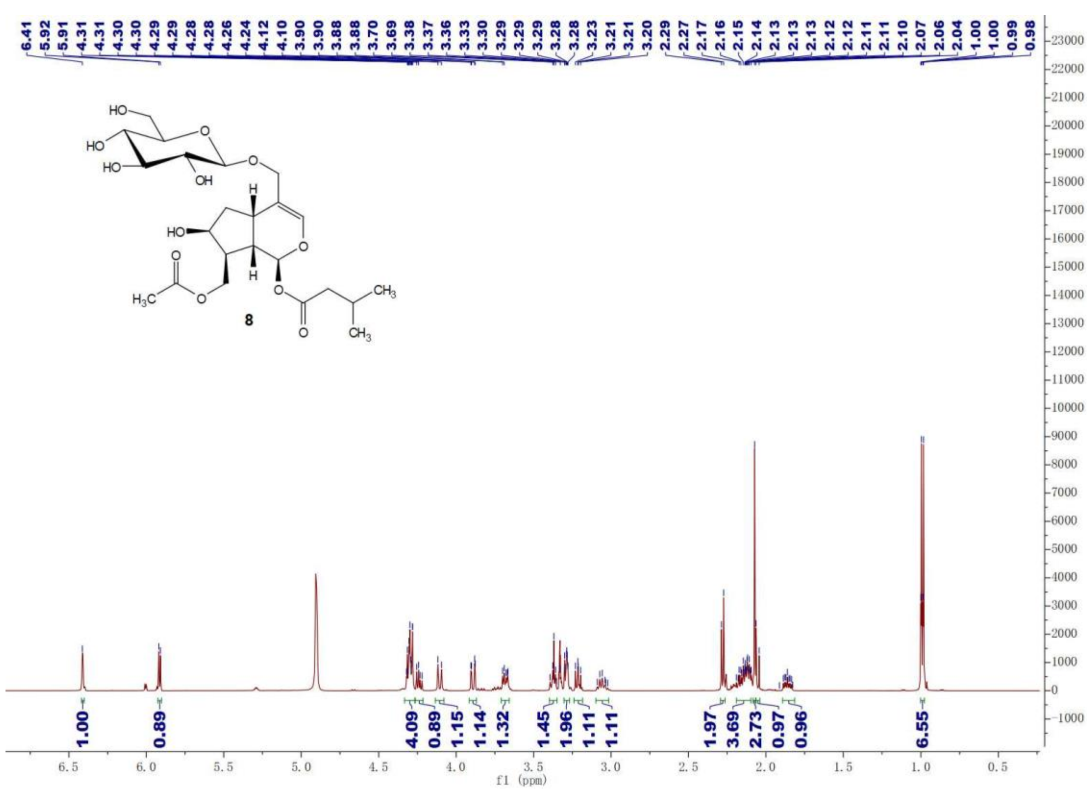

Figure S31. ^{1}H NMR spectrum of compound **8**.

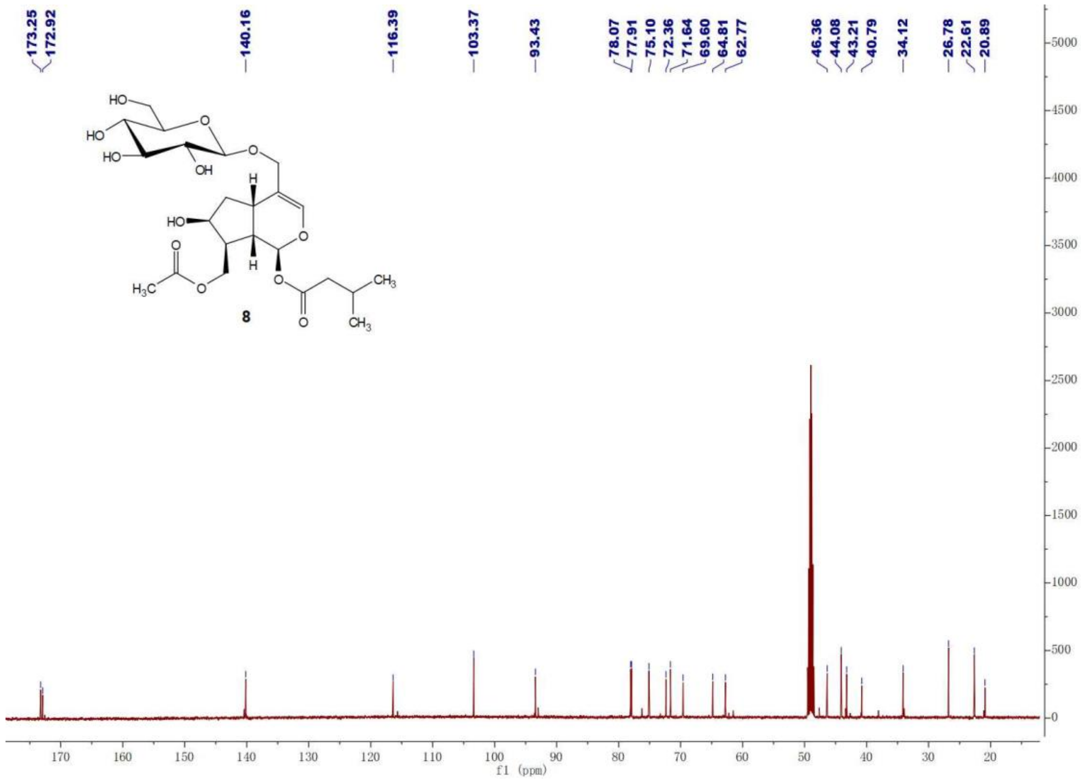

Figure S32. ^{13}C NMR spectrum of compound **8**.

ECD calculations

The conformation search of compound **2** was performed in Molclus (version 1.9.9.4) based on the xtb program by using GFN-xTB method.[1] Conformers with relative energy less than 3 kcal/mol were chosen for further optimization at the B3LYP/6-31g(d) level in gas phase by the Gaussian 09 program.[2] Then 4 conformers were selected for (1*S*,5*S*,7*S*,8*S*,9*S*)-**2** and 6 conformers for (1*R*,5*R*,7*R*,8*R*,9*R*)-**2** based on their Boltzmann distribution. The theoretical calculation of ECD was conducted in MeOH using Time-dependent Density functional theory (TD-DFT) at the B3LYP/6-31+g (d, p) level for the optimized conformers of **2**. Rotatory strengths for a total of 50 excited states were calculated. The low-energy conformers of (1*S*,5*S*,7*S*,8*S*,9*S*)-**2** and (1*R*,5*R*,7*R*,8*R*,9*R*)-**2** were presented in Table S1 and Table S2.

Table S1. B3LYP/6-31g(d) optimized low-energy conformers for (1*S*,5*S*,7*S*,8*S*,9*S*)-**2**

(1*S*,5*S*,7*S*,8*S*,9*S*)-**2-1**	(1*S*,5*S*,7*S*,8*S*,9*S*)-**2-2**
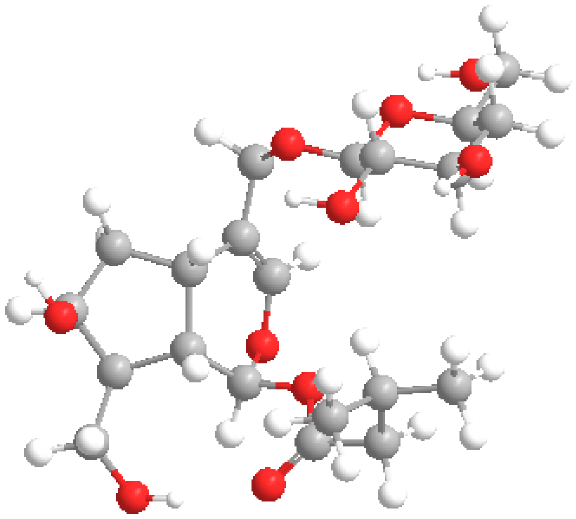 (67.25%)	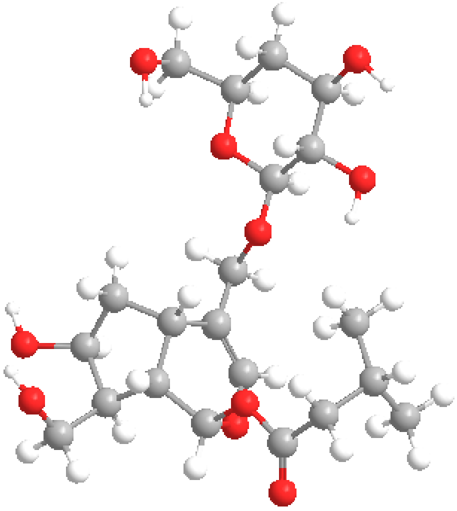 (29.51%)
(1*S*,5*S*,7*S*,8*S*,9*S*)-**2-3**	(1*S*,5*S*,7*S*,8*S*,9*S*)-**2-4**
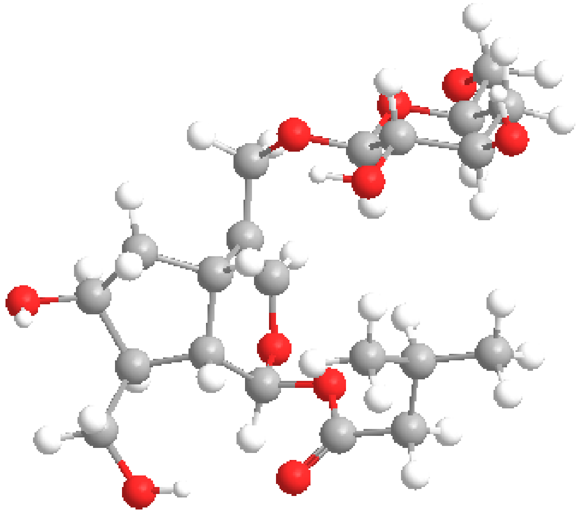 (0.47%)	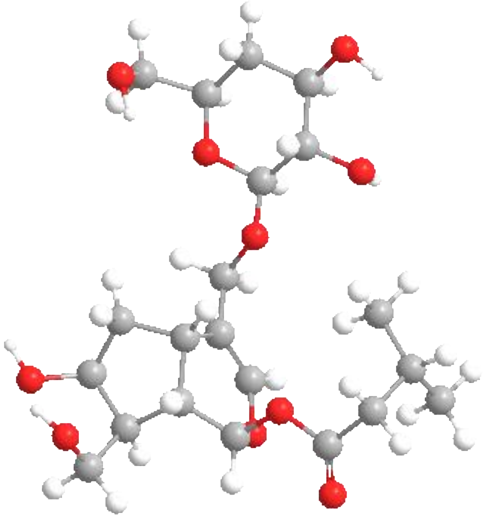 (2.77%)

Table S2. B3LYP/6-31g(d) optimized low-energy conformers for (1*R*,5*R*,7*R*,8*R*,9*R*)-**2**

(1*R*,5*R*,7*R*,8*R*,9*R*)-**2-1**	(1*R*,5*R*,7*R*,8*R*,9*R*)-**2-2**
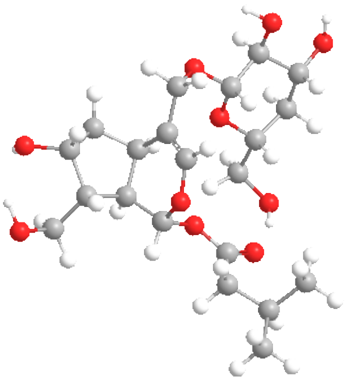 (52.89%)	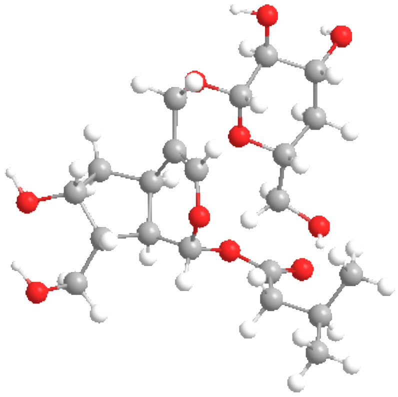 (22.82%)
(1*R*,5*R*,7*R*,8*R*,9*R*)-**2-3**	(1*R*,5*R*,7*R*,8*R*,9*R*)-**2-4**
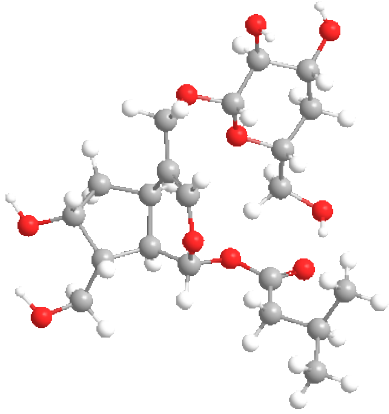 (10.83%)	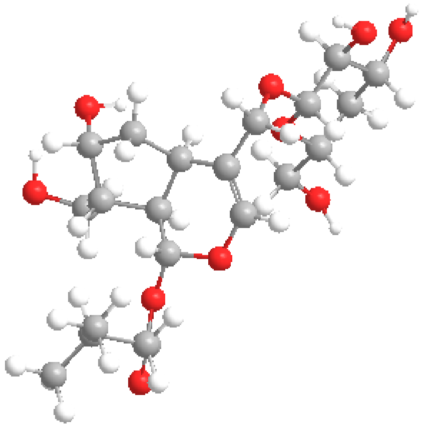 (6.43%)
(1*R*,5*R*,7*R*,8*R*,9*R*)-**2-5**	(1*R*,5*R*,7*R*,8*R*,9*R*)-**2-6**
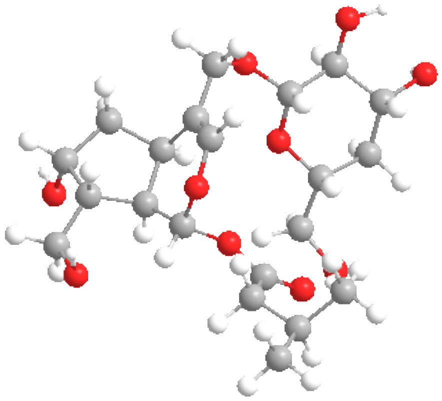 (5.86%)	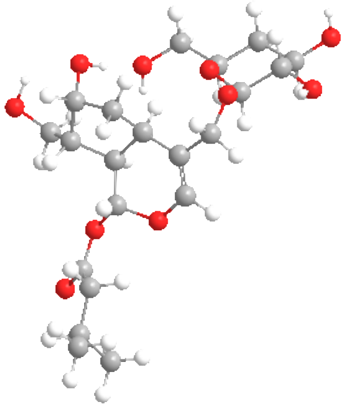 (1.17%)

Table S3. ^{1}H NMR and ^{13}C NMR data of compounds **3~5**.

Position	Compound **3**[a]		Compound **4**[b]		Compound **5**[a]	
	δ_C, type (125 MHz)	δ_H (500 MHz, J in Hz)	δ_C, type (125 MHz)	δ_H (500 MHz, J in Hz)	δ_C, type (150 MHz)	δ_H (600 MHz, J in Hz)
1	176.4, C		177.3, C		68.7, CH_2	4.31 (dd, 11.7, 4.2)
						4.15 (dd, 11.7, 3.3)
2						
3	73.6, CH_2	4.18 (dd, 11.0, 3.5)	72.4, CH_2	4.76 (dd, 12.0, 1.0)	173.6, C	
		3.96 (dd, 11.0, 11.0)		4.71 (d, 12.0)		
4	36.2, CH	1.62 (m)	144.1, C		34.7, CH_2	2.64 (dd, 15.0, 7.3)
						2.37 (dd, 15.0, 3.8)
5	42.5, CH	2.49 (m)	40.9, CH	3.47 (m)	32.8, CH	2.94 (m)
6	41.2, CH_2	2.12 (ddd, 13.5, 7.0, 1.0)	41.3, CH_2	2.15 (ddd, 13.0, 7.5, 1.0)	41.7, CH_2	2.05 (dd, 13.8, 8.1)
		1.39 (ddd, 13.5, 10.5, 3.5)		1.66, (ddd, 13.0, 11.0, 3.5)		1.41 (ddd, 13.8, 10.2, 3.6)
7	74.9, CH	4.44 (t-like, 3.5)	73.4, CH	4.39 (t-like, 4.0)	75.7, CH	4.14 (m)
8	49.4, CH	2.40 (m)	52.0, CH	2.41 (m)	41.8, CH	1.93 (m)
9	41.7, CH	3.10 (dd, 12.0, 9.5)	44.8, CH	3.00 (dd, 12.0, 9.5)	41.6, CH	2.16 (m)
10	62.6, CH_2	4.08 (dd, 11.0, 4.5)	61.8, CH_2	3.87 (dd, 11.0, 5.0)	12.8, CH_3	1.08 (d, 6.9)
		3.74 (dd, 11.0, 5.5)		3.80 (dd, 11.0, 8.5)		
11	15.7, CH_3	1.02 (d, 7.0)	113.7, CH_2	5.19 (dd, 2.0, 1.0)		
				5.07 (dd, 2.0, 1.0)		

[a] measured in $CDCl_3$ [b] measured in CD_3OD

Table S4. ^{1}H NMR (500 MHz) and ^{13}C NMR data (125 MHz) of compounds **6~8**.

Position	Compound **6**[a]		Compound **7**[b]		Compound **8**[a]	
	δ_C, type	δ_H (J in Hz)	δ_C, type	δ_H (J in Hz)	δ_C, type	δ_H (J in Hz)
1	93.4, CH	5.95 (d, 4.9)	93.3, CH	6.59 (d, 5.2)	93.4, CH	5.91 (d, 5.5)
2						
3	138.3, CH	6.28 (s)	139.3, CH	6.57 (br.s)	140.2, CH	6.41 (br.s)
4	119.6, C		116.3, C		116.4, C	
5	33.2, CH	2.99 (q-like)	34.0, CH	3.41 (q-like)	34.1, CH	3.05 (m)
6	40.8, CH_2	2.05 (m)	41.5, CH_2	2.43 (m)	40.8, CH_2	3.89 (dd, 12.0, 2.0)
		1.81 (m)		2.02, (ddd, 12.7, 7.6, 4.8)		3.68 (dd, 12.0,5.5)
7	73.3, CH	4.33 (m)	73.0, CH	4.77 (m)	72.4, CH	4.30 (overlapped)
8	48.9, CH	1.96 (m)	49.0, CH	2.43 (m)	46.4, CH	2.23~2.09 (overlapped)
9	42.9, CH	2.22 (m)	42.6, CH	2.78 (td, 8.5, 5.2)	43.2, CH	2.23~2.09 (overlapped)
10	62.2, CH_2	3.83 (dd, 10.9, 7.3)	62.4, CH_2	4.41 (overlapped)	64.8, CH_2	4.30 (overlapped)
		3.74 (dd, 10.9, 5.7)		4.31 (dd, 10.8, 5.5)		4.30 (overlapped)
11	62.2, CH_2	4.05 (d, 12.5)	69.0, CH_2	4.48 (d, 11.6)	69.6, CH_2	4.24 (dd, 11.0, 6.5)
		3.92 (d, 12.5)		4.21 (overlapped)		4.11 (d, 11.0)
12					173.3, C	
13					20.9, CH_3	2.07 (s)
saccharide moiety						
1'			100.4, CH	4.92 (dd, 9.7, 1.9)	103.4, CH	4.30 (overlapped)
2'			41.1, CH_2	2.57 (ddd, 12.3, 5.1, 1.9)	75.1, CH	3.21 (dd, 9.0, 8.0)

				2.13 (overlapped)		
3'			72.7, CH	4.21 (overlapped)	77.9, CH	3.40~3.27 (overlapped)
4'			73.6, CH	4.08 (t, 9.0)	71.6, CH	3.40~3.27 (overlapped)
5'			79.0, CH	3.83 (ddd, 9.3, 5.3, 2.6)	78.1, CH	3.40~3.27 (overlapped)
6'			63.2, CH_2	4.56 (dd, 11.7, 2.5)	62.8, CH_2	3.68 (dd, 12.0, 5.5)
				4.41 (overlapped)		3.89 (dd, 12.0, 2.0)
isovaleryl moiety						
1''	173.4, C		172.3, C		172.9, C	
2''	44.2, CH_2	2.25 (m)	43.6, CH_2	2.22 (m)	44.1, CH_2	2.28 (d, 7.0)
		2.24 (m)		2.22 (m)		2.28 (d. 7.0)
3''	26.8, CH	2.09 (m)	26.1, CH	2.13 (overlapped)	26.8, CH	2.23~2.09 (overlapped)
4''	22.6, CH_3	0.98 (d, 6.7)	22.7, CH_3	0.87 (d, 6.6)	22.6, CH_3	0.99 (d, 6.5)
5''	22.6, CH_3	0.98 (d, 6.7)	22.7, CH_3	0.87 (d. 6.6)	22.6, CH_3	0.99 (d, 6.5)

[a] measured in CD_3OD [b] measured in C_5D_5N

References

1. (a) Lu T. gau_xtb: A Gaussian interface for xtb code, http://sobereva.com/soft/gau_xtb (accessed May 18th, 2021) (b)Lu T. molclus program, Version 1.9.9.4, http://www.keinsci.com/research/molclus.html (accessed May 18th, 2021)
2. Gaussian 09, Revision E.01, Frisch, M. J.; Trucks, G. W.; Schlegel, H. B.; Scuseria, G. E.; Robb, M. A.; Cheeseman, J. R.; Scalmani, G.; Barone, V.; Mennucci, B.; Petersson, G. A.; Nakatsuji, H.; Caricato, M.; Li, X.; Hratchian, H. P.; Izmaylov, A. F.; Zheng, J.; Sonnenberg, J. L.; Hada, M.; Ehara, M.; Toyota, K.; Fukuda, R.; Hasegawa, J.; Ishida, M.; Nakajima, T.; Honda, Y.; Kitao, O.; Nakai, H.; Vreven, T.; J. A.; Jr.; Peralta, J. E.; Ogliaro, F.; Bearpark, M.; Heyd, J. J.; Brothers, E.; Kudin, K. N.; Staroverov, V. N.; Keith, T.; Kobayashi, R.; Normand, J.; Raghavachari, K.; Rendell, A.; Burant, J. C.; Iyengar, S. S.; Tomasi, J.; Cossi, M.; Rega, N.; Millam, J. M.; Klene, M.; Knox, J. E.; Cross, J. B.; Bakken, V.; Adamo, C.; Jaramillo, J.; Gomperts, R.; Stratmann, R. E.; Yazyev, O.; Austin, A. J.; Cammi, R.; Pomelli, C.; Ochterski, J. W.; Martin, R. L.; Morokuma, K.; Zakrzewski, V. G.; Voth, G. A.; Salvador, P.; Dannenberg, J. J.; Dapprich, S.; Daniels, A. D.; Farkas, O.; Foresman, J. B.; Ortiz, J. V.; Cioslowski, J.; Fox, D. J. Gaussian, Inc., Wallingford CT, 2013.
